# Barriers to Health Care and Cancer Screening

**DOI:** 10.1001/jamanetworkopen.2026.7024

**Published:** 2026-04-14

**Authors:** Aaron A. Gurayah, Anjile An, Manish Kuchakulla, Faith Morley, Daniel M. Markowitz, Jialin Mao, Meenakshi Davuluri, Bashir Al Hussein Al Awamlh, David M. Nanus, Rulla M. Tamimi, Kevin H. Kensler

**Affiliations:** 1 Department of Urology, Weill Cornell Medical Center, New York, New York; 2 Department of Population Health Sciences, Weill Cornell Medical Center, New York, New York; 3 MD Program, Weill Cornell Medicine, New York, New York; 4 Department of Medicine, Weill Cornell Medical Center, New York, New York; 5 Division of Hematology/Oncology, Weill Cornell Medical Center, New York, New York; 6 Sandra and Edward Meyer Cancer Center, Weill Cornell Medical Center, New York, New York

## Abstract

**Importance:**

Identification of health care access-related factors associated with lower rates of cancer screening may help inform targeted interventions to mitigate barriers and ameliorate screening disparities.

**Objective:**

To examine the multifaceted obstacles associated with screening for breast, cervical, colorectal, lung, and prostate cancer in a diverse population.

**Design, Setting, and Participants:**

This cohort study included longitudinal data from the National Institutes of Health’s All of Us (AoU) Research Program (2017-2023). Nonincarcerated individuals aged 18 years or older residing in the US were eligible to participate by enrolling either online or through 1 of approximately 67 health care organizations. Identified participants within the AoU Research Program met US Preventive Services Task Force screening criteria for breast, colorectal, cervical, lung, or prostate cancer. Data were analyzed from October 2024 to January 2026.

**Main Outcomes and Measures:**

Participants self-reported whether 9 potential barriers delayed their medical care in the past year. Multivariable-adjusted odds ratios were estimated for the association between each barrier, the barrier burden, as well as for patterns of interrelated barriers identified using factor analysis and adherence to cancer screening recommendations.

**Results:**

In total, 160 691 participants were eligible for cancer screening including 42 908 participants in the breast (median age at last follow-up, 60 [IQR, 52-67] years; 100% female), 45 791 in the cervical (median age at last follow-up, 46 [IQR, 35-56] years; 100% female), 55 986 in the colorectal (median age at last follow-up, 66 [IQR, 57-73] years; 63% female), 3358 in the lung (median age at last follow-up, 66 [IQR, 59-72] years; 53% female), and 12 648 in the prostate cancer (median age at last follow-up, 63 [IQR, 59-66] years; 100% male) screening cohorts. Out-of-pocket costs, nervousness about seeing clinicians, and inability to get time off work were the most cited barriers. Participants reporting 3 or more barriers to care had significantly lower screening rates compared with those who reported no barriers for all cancer types, ranging from 18% (odds ratio [OR], 0.82; 95% CI, 0.76-0.88) for colorectal cancer to 32% (OR, 0.68; 95% CI, 0.46-0.97) for lung cancer. Three latent factors were consistently identified across cancer sites reflecting cost concerns, logistical barriers (eg, transportation), and competing obligations (eg, time off work). In multivariable analyses, cost concerns were associated with odds of screening for breast cancer (OR, 0.73; 95% CI, 0.66-0.80), cervical cancer (OR, 0.80; 95% CI, 0.73-0.87), and colorectal cancer (OR, 0.85; 95% CI, 0.77-0.94) and logistical barriers for breast cancer (OR, 0.75; 95% CI, 0.63-0.89), cervical cancer (OR, 0.78; 95% CI, 0.65- 0.93), and colorectal cancer (OR, 0.78; 95% CI, 0.65-0.94).

**Conclusions and Relevance:**

In this cohort study of participants eligible for cancer screening, barriers to access and barriers related to cost concerns and logistics were associated with lower screening rates. Policies and interventions must target multiple dimensions of access simultaneously to increase cancer screening.

## Key Points

**Question:**

Are barriers to health care associated with cancer screening rates for breast, cervical, colorectal, lung, and prostate cancers in a diverse population?

**Findings:**

In this cohort study of data from 160 691 adults eligible for cancer screening, out-of-pocket costs, nervousness about seeing clinicians, and inability to get time off work were the most cited barriers. In multivariable analyses, cost concerns and logistical barriers were associated with lower screening rates for breast, cervical, and colorectal cancer.

**Meaning:**

These findings suggest the cumulative burden of barriers to access and those related to cost concerns and logistics are associated with lower screening rates; improved policies and interventions are needed to target multiple dimensions of access simultaneously.

## Teaser

This cohort study assesses the association of barriers to accessing health care, including transportation, cost of care, and competing obligations, with cancer screening rates.

## Introduction

Early detection through routine cancer screening, as recommended by the US Preventive Services Task Force (USPSTF) guidelines, remains one of the most effective strategies for reducing morbidity and mortality associated with common malignant tumors.^1,2,3,4,5^ These recommendations have led to substantial improvements in survival rates when cancers are detected at more treatable stages.^6^ Cancer prevention and screening efforts accounted for 4.75 million deaths averted from 1975 to 2020.^7^

Despite well-established guidelines, substantial disparities persist in screening across various demographic, socioeconomic, and geographic groups.^8^ In 2020, approximately 28 million US residents lacked insurance, limiting access to care and preventive screening.^9^ Studies have shown that 65% to 83% of individuals do not undergo prostate cancer screening while up to 83% do not undergo lung cancer screening.^10,11^ In a study of 100 000 women who completed a cancer screening survey, one-fifth of participants had not undergone routine screening for breast (21.2%), cervical (20%), and colorectal cancer (28.7%).^12^

A complex interplay of individual, systemic, and societal factors contributes to underuse of cancer screening services. Barriers such as limited health literacy and financial constraints are associated with screening participation.^13^ Here, we aimed to comprehensively examine the multifaceted obstacles associated with screening for 5 of the most common cancer types, breast, cervical, colorectal, lung, and prostate cancer.

Unlike prior studies^11,13^ limited by small less diverse samples, the National Institutes of Health (NIH) All of Us (AoU) program provides a large diverse cohort with validated self-reported data. Therefore, we combined objective electronic health record (EHR) data with patient-reported information to identify, quantify, and understand the key obstacles that affect whether individuals undergo cancer screening. Many health care access barriers are correlated, and exploratory factor analysis can identify underlying constructs that capture broader patterns of challenges affecting preventive care, including cancer screening. These findings may help guide clinicians who counsel diverse patients about the cancer screening as well as inform public health initiatives.

## Methods

This cohort study was deemed exempt from review by the Weill Cornell Medical College Institutional Review Board.^14^ Informed consent was waived due to use of deidentified data. The data are made publicly available to registered users through the AoU Researcher Workbench. This study was conducted and reported in accordance with the Strengthening the Reporting of Observational Studies in Epidemiology (STROBE) guideline. Data were analyzed from October 2024 to January 2026.

### Study Population

The NIH AoU Research Program was established in 2017 and contains longitudinal data on US participants, with enriched participation from populations historically underrepresented in research, including individuals from racial and ethnic minority groups, lower-income households, individuals with lower educational attainment, and individuals residing in underserved areas.^15^ Participants enroll digitally through the AoU website or a smartphone app. All nonincarcerated individuals aged 18 years or older residing in the US are eligible to participate by enrolling either online or through 1 of approximately 67 health care organizations. Participants can link their EHR at these participating health care organizations and EHR data are standardized to the Observational Medical Outcomes Partnership Common Data Model. As of the most recent data release (version 8), there are 633 540 participants with follow-up through October 1, 2023.

### Barriers to Health Care Access

After enrollment, participants must complete several baseline surveys, providing information on sociodemographic characteristics, overall health, and personal and family medical history. Once completed, they are then administered a survey about health care access and use (available in English and Spanish).^16^ Within the health care access survey (48% completion rate), participants were asked to report whether any of 9 potential reasons had led them to delay getting medical care during the past 12 months: (1) “didn’t have transportation,” (2) “you live in a rural area where distance to the health care provider is too far,” (3) “you were nervous about seeing a health care provider,” (4) “couldn’t get time off work,” (5) “couldn’t get childcare,” (6) “you provide care to an adult and could not leave him/her,” (7) “couldn’t afford the copay,” (8) “your deductible was too high/or could not afford the deductible,” and (9) “you had to pay out of pocket for some or all of the procedure.” A barrier burden score was calculated for each participant by summing the number of affirmative responses (range 0-9).

### Adherence to Cancer Screening Recommendations

The USPSTF maintains the following grades for these 5 cancer types: breast (grade B), cervical (grade A), colorectal (grade A), lung (grade B), and prostate (grade C).^1,2,3,4,5^ Grade A indicates high certainty of substantial net benefit and warrants routine offering of the service, while grade B reflects high or moderate certainty of substantial net benefit and also supports offering the service, and grade C denotes at least fair evidence of effectiveness but only a small net benefit, for which the service should be offered selectively based on clinical judgment. Due to variation in guidelines during follow-up, the screening guidelines applied reflect the official USPSTF recommendations current as of October 1, 2023, the last day of EHR follow-up in version 8 of the AoU data release.

Receipt of cancer screening was ascertained in the EHR using procedure and laboratory codes summarized in eTable 1 in Supplement 1.^17^ As a condition of inclusion, participants had to have the respective site-specific number of years in their EHR history as the largest interval of the screening guideline. For example, as USPSTF guidelines recommend biennial breast cancer screening, eligible participants must have at least 2 years of follow-up from the earliest documented date to the most recent one in their EHR to adequately ascertain adherence.^5^ Age was defined as the participant’s age at their last EHR encounter during the follow-up period, reflecting the time when screening eligibility and receipt were most recently observable and accommodating variability in timelines of survey completion across participants. Further definitions for site specific adherence vs nonadherence in this study, as well as references to the USPSTF guidelines can be found in eTable 2 in Supplement 1.

### Inclusion and Exclusion Criteria

eFigures 1-5 in Supplement 1 reflect the steps taken to identify each respective cancer screening cohort. Conditions of inclusion required meeting-site specific age guidelines at time of last follow-up, a complete health care access questionnaire, and adequate EHR follow-up to ascertain adherence with screening guidelines. To focus on preventive screening, participants with a prior cancer diagnosis or relevant prior surgery at the site of interest at the start of the cancer screening window were excluded. Participants with new diagnoses of cancer during that window were included and receipt of a screening test prior to that diagnosis was ascertained. Codes used in these exclusions can be found in eTable 1 in Supplement 1.^17,18^ Finally, several EHR sites that reported 0 records of cancer screening were excluded. Across cancer screening cohorts, the most common reasons for exclusion were noncompletion of the health care access and use survey and inadequate follow-up time in the linked EHR.

### Covariates of Interest

Participant age was ascertained as age at date of last encounter in their EHR. Participant sex at birth, race and ethnicity, marital status, household annual income, educational attainment, home ownership, health insurance status, and employment status were all self-reported in surveys. Following the Office of Management and Budget standards,^19^ we combined the 2 self-reported race and ethnicity variables into a single measure. Participants identifying as Hispanic were categorized as Hispanic, while those identifying as Black and not Hispanic were categorized as non-Hispanic Black, and those identifying as White and not Hispanic were categorized as non-Hispanic White. Due to small sample sizes, American Indian or Alaska Native, Asian, Middle Eastern or North African, Native Hawaiian or Other Pacific Islander, and multiracial were grouped as other. Race and ethnicity data were included to fully characterize the cohort. State of residence was grouped by US census region. Finally, we estimated participant smoking pack-year history based on self-reported smoking history.^20^

### Statistical Analysis

Frequency of adherence with cancer screening was evaluated for each individual barrier and the barrier burden using descriptive statistics. Continuous variables are presented as medians with IQR and categorical variables are presented as numbers with percentages. Multivariable logistic regression models were fit to evaluate the associations between the 9 individual barriers and the barrier burden with respect to screening adherence for each cancer site. A minimally adjusted model was fit accounting for age at last follow-up, sex at birth (colorectal and lung only), smoking status (lung only) and pack-year history (lung only). A fully adjusted model was also fit, which included variables in the minimally adjusted model in addition to race and ethnicity, marital status, annual household income, home ownership, census region, health insurance, and employment status. To account for clinical variability and delays in receipt of preventive care, we performed a sensitivity analysis allowing modest flexibility in guideline-recommended screening intervals. Specifically, we expanded the acceptable ascertainment window for screening adherence for an approved screened test by 25% (eg, lung cancer screening was evaluated over a 15-month period rather than a 12-month period). Second, as participants who completed the optional health care access and use survey differed systematically from nonrespondents, we performed a sensitivity analysis to evaluate the potential implications of selection bias. The inverse probability of selection weights was estimated using age, sex, race and ethnicity, income, educational attainment, and census region, and weighted logistic regression with robust SEs was used to assess associations between health care access barriers and cancer screening adherence.

To explore underlying patterns among correlated barriers and evaluate their combined associations with screening, we used exploratory factor analysis to identify latent constructs, which were then included simultaneously in multivariable models.^21,22^ The Kaiser-Meyer-Olkin test was conducted to determine whether there was a sufficient number of significantly correlated items. The sampling adequacy of each item was evaluated, with items with values lower than 0.60 removed due to inadequate correlation with other items. To determine the appropriate number of factors, a scree plot graphing the number of factors against their eigenvalues was used. Patients were then categorized into barrier factor groups using the items loaded to each factor, with patients who did not fall into any of the barrier factor categories classified as no or minimal barriers. The barrier factor category variable was then used in subsequent multivariable logistic regression models for adherence to screening recommendations. All *P* values we considered significant at α =.05. Analyses were conducted using R version 4.4.0 (R Foundation for Statistical Computing).

## Results

A total of 160 691 participants were included. Characteristics of participants who were eligible for cancer screening are shown in Table 1, including 42 908 participants in the breast (median age at last follow-up, 60 [IQR, 52-67] years; 100% female), 45 791 in the cervical (median age at last follow-up, 46 [IQR, 35-56] years; 100% female), 55 986 in the colorectal (median age at last follow-up, 66 [IQR, 57-73] years; 63% female; 36% male), 3358 in the lung (median age at last follow-up, 66 [IQR, 59-72] years; 53% female; 46% male), and 12 648 in the prostate cancer (median age at last follow-up, 63 [IQR, 59-66] years; 100% male) screening cohorts. In the colorectal cancer screening cohort, 7% were Hispanic, 10% were non-Hispanic Black, 77% were non-Hispanic White, 6% identified as other (American Indian or Alaska Native, Asian, Middle Eastern or North African, Native Hawaiian or Other Pacific Islander, and multiracial), and 12% had an annual income of $25 000 or less, and 13% had a high school education or less. Participant characteristics within each screening cohort stratified by the health care access barrier burden are shown in eTables 3-7 in Supplement 1. Patterns were similar across cancer screening cohorts, but within the colorectal cancer screening cohort, participants reporting 3 or more barriers tended to be younger (median age 58 [IQR, 51-64] years vs 67 [IQR, 59-74] years), female (75% vs 60%), Hispanic (11% vs 6%), non-Hispanic Black (16% vs 8%) compared with those reporting no health care access barriers. Similarly, participants reporting 3 or more barriers were less likely to be married or living with a partner (50% vs 68%), have an advanced degree (19% vs 37%), and own their home (55% vs 79%) compared with participants reporting no barriers. Participants reporting 3 or more barriers were more likely to be out of work or unable to work (27% vs 12%), have Medicaid insurance (14% vs 8%), or no health insurance (7% vs 1%).

**Table 1.** Characteristics of All of Us Research Program Participants Eligible for Breast, Cervical, Colorectal, Lung, and Prostate Cancer Screening^a^

Characteristic	Cancer screening, No. (%)				
	Breast (n = 42 908)	Cervical (n = 45 791)	Colorectal (n = 55 986)	Lung (n = 3358)	Prostate (n = 12 648)
Age at last follow up, median (IQR), y	60 (52-67)	46 (35-56)	66 (57-73)	66 (59-72)	63 (59-66)
Sex at birth					
Female	42 908 (100)	45 791 (100)	35 208 (63)	1796 (53)	NA
Male	NA	NA	20 356 (36)	1532 (46)	12 648 (100)
Other or unknown	NA	NA	422 (1)	30 (1)	NA
Race and ethnicity^b^					
Hispanic	4442 (10)	29 716 (65)	3932 (7)	143 (4)	1267 (10)
Non-Hispanic Black	4899 (11)	7324 (16)	5333 (10)	275 (8.2)	1170 (9)
Non-Hispanic White	30 800 (72)	4863 (11)	43 166 (77)	2777 (83)	9351 (74)
Other race or ethnicity^c^	1708 (4)	2927 (6)	2078 (4)	72 (2)	527 (4)
Unknown	1059 (3)	961 (2)	1477 (3)	91 (3)	333 (3)
Marital status					
Divorced/separated/widowed	11 301 (26)	7445 (16)	13 055 (23)	1207 (36)	2330 (18)
Married/living with partner	25 665 (60)	26 290 (57)	36 021 (64)	1683 (50)	8553 (68)
Never married	5443 (13)	11 426 (25)	6302 (11)	435 (13)	1609 (13)
Unknown	499 (1)	630 (1)	608 (1)	33 (1)	156 (1)
Annual income, $					
0-24 999	6576 (15)	7986 (17)	6654 (12)	848 (25)	1968 (16)
25 000-49 999	6216 (14)	7332 (16)	7602 (14)	749 (22)	1378 (11)
50 000-74 999	5833 (14)	5807 (13)	7706 (14)	518 (15)	1358 (11)
75 000-99 999	5103 (12)	4901 (11)	6955 (12)	383 (11)	1301 (10)
100 000 or greater	13 917 (32)	14 252 (31)	20 578 (37)	546 (16)	5133 (41)
Unknown	5263 (12)	5513 (12)	6491 (12)	314 (9)	1510 (12)
Educational attainment					
High school or less	6438 (15)	7679 (17)	7170 (13)	741 (22)	2157 (17)
Some college	11 752 (27)	12 209 (27)	13 578 (24)	1415 (42)	3128 (25)
College	11 949 (28)	13 677 (30)	15 748 (28)	683 (20)	3381 (27)
Advanced degree	12 256 (29)	11 665 (25)	18 879 (34)	477 (14)	3840 (30)
Unknown	513 (1)	561 (1)	611 (1)	42 (1)	142 (1)
Home ownership					
Own	29 733 (69)	24 073 (53)	42 043 (75)	2058 (61)	8826 (70)
Rent	10 354 (24)	16 826 (37)	11 099 (20)	1011 (30)	2927 (23)
Other/unknown	2821 (7)	4892 (11)	2844 (5)	289 (8)	895 (7)
Health insurance					
Self/employer purchased	22 635 (53)	29 997 (66)	23 681 (42)	938 (28)	6448 (51)
Medicare	11 728 (27)	2779 (6)	23 485 (42)	1534 (46)	3025 (24)
Medicaid	5310 (12)	8622 (19)	4316 (8)	515 (15)	1436 (11)
Military/VA	898 (2)	983 (2)	1894 (3)	171 (5)	867 (7)
Other	972 (2)	1220 (3)	1006 (2)	76 (2)	273 (2)
None	1015 (2)	1747 (4)	1096 (2)	94 (3)	474 (4)
Unknown	350 (1)	443 (1)	508 (1)	30 (1)	125 (1)
Employment status					
Employed	22 809 (53)	32 043 (70)	24 869 (44)	1144 (34)	6607 (52)
Retired	10 523 (25)	1519 (3)	22 302 (40)	1351 (40)	3507 (28)
Unable/out of work	7001 (16)	7661 (17)	6737 (12)	786 (23)	2336 (18)
Other	2575 (6)	4568 (10)	2078 (4)	77 (2)	198 (2)
Census region of residence					
Northeast	13 619 (32)	14 100 (31)	20 602 (37)	1137 (34)	3546 (28)
Midwest	16 212 (38)	15 367 (34)	22 333 (40)	1209 (36)	4173 (33)
South	5318 (12)	6226 (14)	6015 (11)	354 (11%)	1839 (15)
West	<7750 (18)	<10 090 (22)	7004 (13)	<660 (20)	<3090 (24)
Other	<20 (<1)	<20 (<1)	32 (<1)	<20 (<1)	<20 (<1)
Smoking status^d^					
Current	NA	NA	NA	968 (29)	NA
Former	NA	NA	NA	2390 (71)	NA
Smoking pack-year history, median (IQR)^d^	NA	NA	NA	37 (26-50)	NA

Abbreviations: NA, not applicable; VA, Veterans Affairs.

^a^ Percentages may not total 100 due to rounding or suppression of small participant counts. Per the data and statistics dissemination policy of the All of Us Research Program, all participant counts less than 20 individuals have been suppressed.

^b^ Participant race and ethnicity were self-reported in surveys.

^c^ Other race and ethnicity includes participants who self-identified as non-Hispanic American Indian or Alaska Native, non-Hispanic Asian or Pacific Islander, non-Hispanic Middle Eastern or North African, or as belonging to 2 or more racial groups.

^d^ Cigarette smoking history variables were ascertained in the lung cancer screening cohort only.

Across cancer screening cohorts, the most commonly cited barrier to health care was concerns about out-of-pocket costs, with 13% (colorectal, prostate) to 21% (cervical) of screening-eligible participants stating that they had delayed seeking care for this reason in the past year (eFigure 6, eTable 7 in Supplement 1). Additional concerns about health insurance, including being able to afford the deductible (ranging from 7% in prostate cancer to 14% in cervical cancer) and copay (ranging from 5.4% in colorectal cancer to 12% in cervical cancer) were commonly cited barriers. Overall, 37% of breast, 50% of cervical, 28% of colorectal, 36% of lung, and 28% of prostate cancer screening-eligible participants reported that at least 1 of these factors had led them to delay receiving health care in the prior year.

Overall, screening adherence rates were as follows: breast, 52%; cervical, 29%; colorectal, 54%; lung, 16%; and prostate, 44%. Participants who were adherent with screening recommendations were less likely to report experiencing each of the 9 potential barriers (eFigure 6 in Supplement 1). Moreover, for each cancer type, the proportion of participants adherent with screening recommendations decreased with increasing number of barriers experienced (Figure), with adherence rates of 44% for breast, 24% for cervical, 39% for colorectal, 10% for lung, and 35% for prostate cancer among participants reporting 3 or more barriers.

**Figure.**
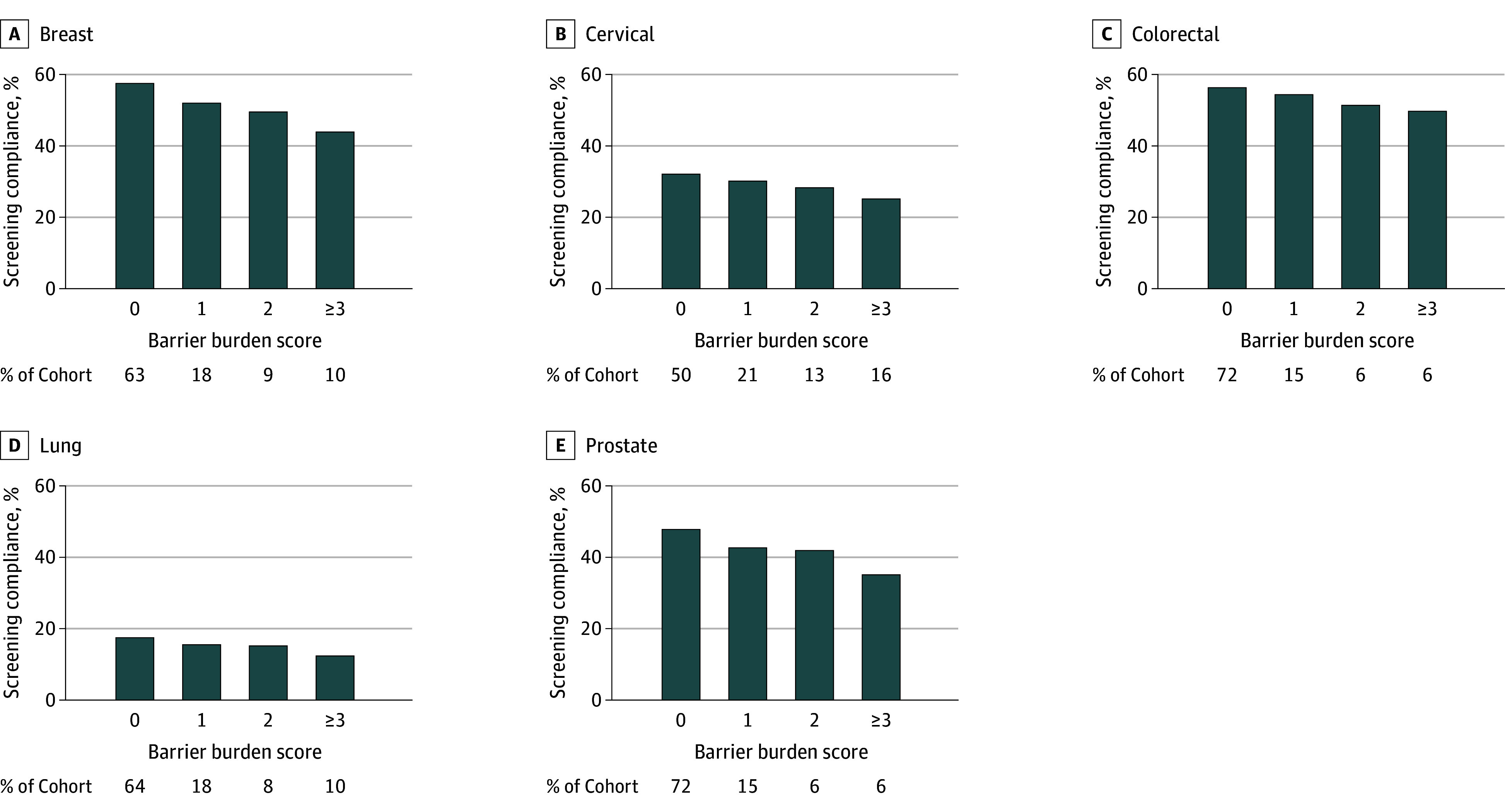
Bar Graph of Proportion of All of Us Participants Adherent With US Preventive Services Task Force Cancer Screening Recommendations by Health Care Access Barrier Burden Score The barrier burden score was calculated for each participant by summing the number of affirmative responses (range 0-9).

In age-adjusted and multivariable-adjusted models, the health care access barrier burden was inversely associated with adherence to cancer screening recommendations (Table 2). The odds ratios (ORs) modestly attenuated when accounting for participant sociodemographic characteristics, and in the fully adjusted model, individuals reporting 3 or more barriers were 29% less likely to be adherent to breast cancer screening recommendations (OR, 0.71; 95% CI, 0.66-0.76), 20% less likely for cervical cancer (OR, 0.80; 95% CI, 0.75-0.85), 18% less likely for colorectal cancer (OR, 0.82; 95% CI, 0.76-0.88), 32% less likely for lung cancer (OR, 0.68; 95% CI, 0.46-0.97), and 26% less likely for prostate cancer (OR, 0.74; 95% CI, 0.63-0.87). Nervousness about seeing a health care clinician, lack of transportation, and concerns about copays, deductibles, and out-of-pocket costs were consistently independently associated with lower odds of cancer screening recommendation adherence across cancer types. Findings were similar when extending screening intervals by 25% to ascertain adherence to screening recommendations (eTable 8 in Supplement 1) and when using inverse probability of selection weighting to account for differences between participants who did and did not complete the health care access survey (eTable 9 in Supplement 1).

**Table 2.** Adherence With USPSTF Cancer Screening Recommendations by Self-Reported Barriers to Health Care and Barrier Burden

Reason for delaying care^a^	OR (95% CI)									
	Breast		Cervical		Colorectal		Lung		Prostate	
	Model 1^b^	Model 2^c^	Model 1^b^	Model 2^c^	Model 1^b^	Model 2^c^	Model 1^b^	Model 2^c^	Model 1^b^	Model 2^c^
Barrier burden score^d^										
0	[Reference]	[Reference]	[Reference]	[Reference]	[Reference]	[Reference]	[Reference]	[Reference]	[Reference]	[Reference]
1	0.81 (0.77-0.86)	0.86 (0.82-0.91)	0.93 (0.88-0.98)	0.96 (0.91-1.02)	0.93 (0.89-0.98)	0.94 (0.89-0.99)	0.90 (0.70-1.15)	0.87 (0.67-1.13)	0.83 (0.75-0.91)	0.90 (0.81-0.99)
2	0.75 (0.70-0.80)	0.81 (0.76-0.87)	0.84 (0.78-0.89)	0.88 (0.82-0.94)	0.84 (0.79-0.90)	0.84 (0.78-0.90)	0.91 (0.64-1.28)	0.91 (0.62-1.29)	0.80 (0.69-0.93)	0.89 (0.77-1.04)
≥3	0.60 (0.56-0.64)	0.71 (0.66-0.76)	0.71 (0.66-0.75)	0.80 (0.75-0.85)	0.79 (0.74-0.85)	0.82 (0.76-0.88)	0.75 (0.52-1.06)	0.68 (0.46-0.97)	0.62 (0.53-0.72)	0.74 (0.63-0.87)
Nervous about seeing health care provider	0.74 (0.69-0.78)	0.78 (0.74-0.83)	0.83 (0.78-0.87)	0.87 (0.82-0.92)	0.92 (0.87-0.98)	0.93 (0.87-0.99)	0.90 (0.65-1.21)	0.87 (0.63-1.19)	0.81 (0.71-0.93)	0.89 (0.77-1.02)
Couldn’t get childcare	0.92 (0.80-1.06)	1.03 (0.89-1.19)	0.87 (0.80-0.95)	1.00 (0.91-1.09)	0.94 (0.79-1.12)	1.03 (0.87-1.23)	1.55 (0.51-3.95)	1.55 (0.55-4.06)	0.56 (0.31-0.97)	0.70 (0.38-1.23)
Live in rural area and distance is too far	0.55 (0.49-0.61)	0.72 (0.64-0.80)	0.61 (0.55-0.68)	0.77 (0.69-0.86)	0.71 (0.64-0.80)	0.77 (0.69-0.86)	0.83 (0.50-1.30)	0.84 (0.50-1.34)	0.75 (0.61-0.92)	0.92 (0.74-1.15)
Provide care to an adult and could not leave him/her	0.72 (0.64-0.81)	0.87 (0.77-0.98)	0.70 (0.61-0.80)	0.87 (0.75-0.99)	0.85 (0.75-0.95)	0.90 (0.80-1.02)	0.54 (0.24-1.06)	0.60 (0.26-1.20)	0.72 (0.54-0.95)	0.85 (0.65-1.13)
Couldn’t get time off work	0.96 (0.90-1.03)	0.90 (0.84-0.97)	0.99 (0.93-1.04)	0.94 (0.89-1.00)	0.96 (0.89-1.03)	0.93 (0.86-1.00)	0.69 (0.42-1.07)	0.64 (0.39-1.02)	0.80 (0.67-0.95)	0.82 (0.69-0.98)
Didn’t have transportation	0.55 (0.51-0.59)	0.80 (0.74-0.87)	0.66 (0.62-0.71)	0.91 (0.84-0.98)	0.83 (0.77-0.89)	0.91 (0.84-0.99)	0.82 (0.58-1.13)	0.80 (0.56-1.12)	0.60 (0.52-0.70)	0.79 (0.68-0.93)
Couldn’t afford the copay	0.66 (0.62-0.71)	0.80 (0.74-0.86)	0.76 (0.71-0.81)	0.85 (0.80-0.91)	0.89 (0.83-0.96)	0.93 (0.86-1.00)	0.85 (0.59-1.19)	0.79 (0.54-1.14)	0.72 (0.62-0.85)	0.87 (0.73-1.02)
Deductible was too high	0.78 (0.73-0.83)	0.81 (0.76-0.87)	0.86 (0.81-0.91)	0.86 (0.81-0.92)	0.86 (0.81-0.92)	0.84 (0.78-0.90)	0.85 (0.60-1.18)	0.82 (0.57-1.16)	0.76 (0.66-0.87)	0.83 (0.72-0.96)
Had to pay out of pocket for some/all of procedure	0.77 (0.73-0.81)	0.79 (0.75-0.83)	0.86 (0.81-0.90)	0.86 (0.81-0.90)	0.86 (0.82-0.90)	0.84 (0.80-0.89)	0.89 (0.69-1.15)	0.86 (0.66-1.12)	0.86 (0.78-0.96)	0.90 (0.81-1.00)

Abbreviation: OR, odds ratio; USPSTF, US Preventive Services Task Force.

^a^ Reasons presented verbatim from survey.

^b^ Model 1 includes age at last follow-up, sex at birth (colorectal and lung only), smoking status (current vs former; lung only), pack-year history (lung only).

^c^ Model 2 includes age at last follow-up, sex at birth (colorectal and lung only), smoking status (current vs former; lung only), pack-year history (lung only), self-identified race and ethnicity, annual income, educational attainment, employment status, health insurance status and type, marital status, and census region of residence.

^d^ The barrier burden score was calculated for each participant by summing the number of affirmative responses (range 0-9).

Exploratory factor analysis identified 3 consistent latent barrier factors across all cancer sites except lung: (1) relating to cost concerns (out-of-pocket costs, insurance copays and deductibles), (2) relating to physical barriers (distance to care and transportation), and (3) relating to competing obligations (work, childcare, adult care). For lung cancer, only the first latent factor was identified. Individuals reporting cost concerns were less likely to be adherent with breast (OR, 0.73; 95% CI, 0.66-0.80), cervical (OR, 0.80; 95% CI, 0.73-0.87), and colorectal cancer (OR, 0.85; 95% CI, 0.77-0.94) screening guidelines compared with those reporting no or minimal barriers (Table 3). Physical barriers were similarly associated with lower adherence to breast (OR, 0.75; 95% CI, 0.63-0.89), cervical (OR, 0.78; 95% CI, 0.65-0.93), and colorectal (OR, 0.78; 95% CI, 0.65-0.94) cancer screening guidelines.

**Table 3.** Exploratory Factor Analysis in USPSTF Cancer Screening Recommendations by Barrier

Health care access barrier^a^	Factor loading	OR (95% CI) compared with no/minimal barriers^b^
**Breast cancer**		
Factor 1		
Deductible was too high	0.9	0.73 (0.66-0.80)
Couldn’t afford the copay	0.7	
Had to pay out of pocket for some/all of procedure	0.7	
Factor 2		
Didn’t have transportation	0.7	0.75 (0.63-0.89)
Live in rural area and distance is too far	0.4	
Factor 3		
Couldn’t get time off work	0.4	0.96 (0.74-1.26)
Couldn’t get childcare	0.3	
**Cervical cancer**		
Factor 1		
Deductible was too high	0.9	0.80 (0.73-0.87)
Had to pay out of pocket for some/all of procedure	0.7	
Couldn’t afford the copay	0.7	
Factor 2		
Didn’t have transportation	0.6	0.78 (0.65-0.93)
Live in rural area and distance is too far	0.4	
Factor 3		
Couldn’t get time off work	0.5	0.96 (0.90-1.02)
**Colorectal cancer**		
Factor 1		
Deductible was too high	0.9	0.85 (0.77-0.94)
Couldn’t afford the copay	0.7	
Had to pay out of pocket for some/all of procedure	0.6	
Factor 2		
Didn’t have transportation	0.7	0.78 (0.65-0.94)
Live in rural area and distance is too far	0.4	
Factor 3		
Couldn’t get time off work	0.4	0.89 (0.65-1.21)
Couldn’t get childcare	0.3	
**Lung cancer**		
Factor 1		
Deductible was too high	0.9	0.95 (0.60-1.46)
Couldn’t afford the copay	0.7	
Had to pay out of pocket for some/all of procedure	0.7	
**Prostate cancer**		
Factor 1		
Deductible was too high	0.9	0.89 (0.72-1.09)
Couldn’t afford the copay	0.7	
Had to pay out of pocket for some/all of procedure	0.6	
Factor 2		
Didn’t have transportation	0.7	0.83 (0.58-1.17)
Live in rural area and distance is too far	0.4	
Factor 3		
Couldn’t get childcare	0.4	0.51 (0.14-1.50)
Provide care to an adult and could not leave him/her	0.3	
Couldn’t get time off work	0.3	

Abbreviations: OR, odds ratio; USPSTF, US Preventive Services Task Force.

^a^ Exploratory factor analysis identified 3 consistent latent barrier factors across all cancer sites except lung: (1) relating to cost concerns (out-of-pocket costs, insurance copays and deductibles), (2) relating to physical barriers (distance to care and transportation), and (3) relating to competing obligations (work, childcare, adult care). Reasons are presented verbatim from survey.

^b^ OR is relative to individuals reporting no or minimal barriers and is adjusted for age at last follow-up, sex at birth (colorectal and lung only), smoking status (current vs former; lung only), pack-year history (lung only), self-identified race/ethnicity, annual income, educational attainment, employment status, health insurance status and type, marital status, and census region of residence.

## Discussion

This study offers insights into the association between barriers to health care access and screening across 5 major cancer types: breast, cervical, colorectal, lung, and prostate. The findings in fully adjusted models suggest a modest attenuation with sociodemographic factors and screening adherence alongside structural and logistical barriers. Screening adherence was low overall (ranging from 16% for lung cancer to 54% for colorectal cancer). Low lung cancer screening adherence likely reflects limited awareness, fear or stigma, logistical challenges, and patient-reported financial and access barriers.^23^

Participants facing 3 or more health care access barriers had significantly lower screening odds across all cancer types, with the largest decrease in lung cancer (32% lower). Our study consistently found that financial burdens were associated with reduced odds of screening across all 5 cancer types. Consistent with our item-level findings, exploratory factor analysis revealed financial barriers as the most prominent underlying dimension. As the Affordable Care Act requires most private and public insurers to cover guideline-recommended cancer screening tests without cost-sharing,^24^ reported financial barriers may reflect perceived rather than structural obstacles to screening. Limited awareness of insurance coverage, requirements for preauthorization, and concerns about unexpected costs may contribute to cost-related screening delays, even when the screening test itself is covered.^25,26,27^ Financial hardships can manifest in indirect ways, such as lost wages from taking time off work.^25^ These findings identify cost as a major barrier to preventive care, particularly among populations with low income.^28,29,30,31^ To address screening barriers related to financial constraints, policy initiatives should continue to expand insurance coverage for individuals and subsidize cancer screening programs.^32^ Enhancing access through telehealth may alleviate challenges for those with limited transportation options^33,34^ while community-based educational initiatives can help reduce emotional and social barriers.^35^

In addition to financial constraints, factor analysis suggested that logistical challenges, such as rural residence and limited transportation access, were deterrents to screening. These findings align with existing evidence that individuals in geographically underserved areas experience lower screening rates.^36^ To address logistical barriers, colorectal cancer screening is widely available through mail-based stool testing, while mail-based cervical cancer screening is an emerging approach.^37,38^ In addition, mobile health screening clinics may bring services directly to underserved communities.^39^ In terms of addressing time constraints from work, cities have proposed legislation to provide workers with 5 hours annually to obtain preventive medical care, including screening.^40^

Factors such as nervousness about seeing a clinician and competing responsibilities, such as work or caregiving for a family member, were highlighted in our study. These findings are consistent with previous studies, which have shown that individuals avoid screening due to fear, not only of the procedure itself but also of receiving a potential cancer diagnosis.^41^ Additionally, cultural beliefs that frame cancer as a fatalistic disease can further discourage individuals from seeking screening.^42^ Our findings highlight that individuals facing multiple overlapping barriers are particularly vulnerable to being underscreened and underscores the need for interventions that simultaneously address these overlapping barriers. Future studies should also explore how sociodemographic factors may modify the role of perceived health care barriers in cancer screening, particularly within individual cancer types.

### Limitations

This study has several limitations. First, this is a retrospective analysis using EHR data, which may be subject to documentation inaccuracies, missing data, and sampling biases. This may contribute to the variance in cancer screening rates seen in our study compared with 2023 national benchmarks (study: 52% for breast, 29% for cervical, and 54% for colorectal cancer screening vs national: 80%, 76%, 42%, respectively).^43^ Screening status was ascertained from structured EHR data, which may be incomplete, potentially leading to misclassification or underrecognition of cancer screening.^17^ AoU only provides EHR data from partnering institutions, and thus we were unable to assess whether participants received screening at other facilities. The health care access and use survey had only been completed by 48% of AoU participants, although our findings were similar when using inverse probability of selection weights to assess potential selection biases. Barriers were assessed using general measures of delayed health care rather than screening-specific items, although they likely reflect structural and logistical constraints relevant to cancer screening. Barriers were also measured at a single time point and may change over time with shifts in health status, insurance, and other circumstances.

## Conclusions

In this cohort study of screening-eligible adults, a greater cumulative burden of access, cost, and logistical barriers was associated with lower adherence to cancer screening guidelines. In this large diverse US sample, these barrier domains were independently associated with lower screening rates and had overlapping effects when experienced concurrently. Policies and interventions should address multiple dimensions of access to improve screening rates.

## Notes

Supplement 1. **eTable 1.** Codes utilized within the electronic health record to ascertain receipt of cancer screening, prior cancer diagnoses, and site-specific resections **eTable 2.** Participant characteristics for the breast cancer screening cohort stratified by healthcare access barrier burden **eTable 3.** Participant characteristics for the cervical cancer screening cohort stratified by healthcare access barrier burden **eTable 4.** Participant characteristics for the colorectal cancer screening cohort stratified by healthcare access barrier burden **eTable 5.** Participant characteristics for the lung cancer screening cohort stratified by healthcare access barrier burden **eTable 6.** Participant characteristics for the prostate cancer screening cohort stratified by healthcare access barrier burden **eTable 7.** Proportion of All of Us participants eligible for cancer screening who responded that this barrier had led them to delay receiving healthcare **eTable 8.** Multivariable-adjusted odds ratios (OR) and 95% confidence intervals (95% CI) for adherence with USPSTF cancer screening recommendations by self-reported barriers to healthcare and barrier burden using 25% extended time windows for assessment of screening adherence **eTable 9.** Multivariable-adjusted odds ratios (OR) and 95% confidence intervals (95% CI) for adherence with USPSTF cancer screening recommendations by self-reported barriers to healthcare and barrier burden applying inverse probability of selection weights **eFigure 1.** Flowchart for identification of the breast cancer screening cohort **eFigure 2.** Flowchart for identification of the cervical cancer screening cohort **eFigure 3.** Flowchart for identification of the colorectal cancer screening cohort **eFigure 4.** Flowchart for identification of the lung cancer screening cohort **eFigure 5.** Flowchart for identification of the prostate cancer screening cohort **eFigure 6.** Proportion of All of Us participants reporting that a barrier has led them to delay receiving care in the past year by cancer screening cohort and adherence with USPSTF guidelines within cohort

Supplement 2.
Data Sharing Statement
