# Supplementary material for: Barriers to Health Care and Cancer Screening
Source: JAMA Netw Open. 2026 Apr 14;9(4):e267024. doi: 10.1001/jamanetworkopen.2026.7024 (PMC13080545; doi:10.1001/jamanetworkopen.2026.7024)
Supplement: Supplement 1. — eTable 1. Codes utilized within the electronic health record to ascertain receipt of cancer screening, prior cancer diagnoses, and site-specific resections eTable 2. Participant characteristics for the breast cancer screening cohort stratified by healthcare access barrier burden eTable 3. Participant characteristics for the cervical cancer screening cohort stratified by healthcare access barrier burden eTable 4. Participant characteristics for the colorectal cancer screening cohort stratified by healthcare access barrier burden eTable 5. Participant characteristics for the lung cancer screening cohort stratified by healthcare access barrier burden eTable 6. Participant characteristics for the prostate cancer screening cohort stratified by healthcare access barrier burden eTable 7. Proportion of All of Us participants eligible for cancer screening who responded that this barrier had led them to delay receiving healthcare eTable 8. Multivariable-adjusted odds ratios (OR) and 95% confidence intervals (95% CI) for adherence with USPSTF cancer screening recommendations by self-reported barriers to healthcare and barrier burden using 25% extended time windows for assessment of screening adherence eTable 9. Multivariable-adjusted odds ratios (OR) and 95% confidence intervals (95% CI) for adherence with USPSTF cancer screening recommendations by self-reported barriers to healthcare and barrier burden applying inverse probability of selection weights eFigure 1. Flowchart for identification of the breast cancer screening cohort eFigure 2. Flowchart for identification of the cervical cancer screening cohort eFigure 3. Flowchart for identification of the colorectal cancer screening cohort eFigure 4. Flowchart for identification of the lung cancer screening cohort eFigure 5. Flowchart for identification of the prostate cancer screening cohort eFigure 6. Proportion of All of Us participants reporting that a barrier has led them to delay receiving care in the past year by cancer s [file jamanetwopen-e267024-s001.pdf]

Supplemental Online Content

Gurayah AA, An A, Kuchakulla M. Barriers to health care and cancer screening. *JAMA Netw Open*. 2026;9(4): e267024. doi: 10.1001/jamanetworkopen.2026.7024

eTable 1. Codes utilized within the electronic health record to ascertain receipt of cancer screening, prior cancer diagnoses, and site-specific resections

eTable 2. Participant characteristics for the breast cancer screening cohort stratified by healthcare access barrier burden

eTable 3. Participant characteristics for the cervical cancer screening cohort stratified by healthcare access barrier burden

eTable 4. Participant characteristics for the colorectal cancer screening cohort stratified by healthcare access barrier burden

eTable 5. Participant characteristics for the lung cancer screening cohort stratified by healthcare access barrier burden

eTable 6. Participant characteristics for the prostate cancer screening cohort stratified by healthcare access barrier burden

eTable 7. Proportion of All of Us participants eligible for cancer screening who responded that this barrier had led them to delay receiving healthcare

eTable 8. Multivariable-adjusted odds ratios (OR) and 95% confidence intervals (95% CI) for adherence with USPSTF cancer screening recommendations by self-reported barriers to healthcare and barrier burden using 25% extended time windows for assessment of screening adherence

eTable 9. Multivariable-adjusted odds ratios (OR) and 95% confidence intervals (95% CI) for adherence with USPSTF cancer screening recommendations by self-reported barriers to healthcare and barrier burden applying inverse probability of selection weights

eFigure 1. Flowchart for identification of the breast cancer screening cohort

eFigure 2. Flowchart for identification of the cervical cancer screening cohort

eFigure 3. Flowchart for identification of the colorectal cancer screening cohort

eFigure 4. Flowchart for identification of the lung cancer screening cohort

eFigure 5. Flowchart for identification of the prostate cancer screening cohort

eFigure 6. Proportion of All of Us participants reporting that a barrier has led them to delay receiving care in the past year by cancer screening cohort and adherence with USPSTF guidelines within cohort

© 2026 Gurayah AA et al. *JAMA Network Open.*

This supplemental material has been provided by the authors to give readers additional information about their work.

Supplemental Table 1. Codes utilized within the electronic health record to ascertain receipt of cancer screening, prior cancer diagnoses, and site-specific resections

**CANCER SCREENING**		
Site	Code Type	Codes
Breast Cancer Screening	CPT (Mammogram/US)	77063, 77067, 77057, 76092, 77052, 76642, 76641
	ICD-10	Z12.31
	ICD-9	V76.11, V76.12
	HCPCS (G-Codes)	G9900, G9899
Cervical Cancer Screening	LOINC	10524-7, 18500-9, 19762-4, 19764-0, 19765-7, 19766-5, 19774-9, 33717-0, 47527-7, 47528-5, 11083-3, 12223-4, 14503-7, 14504-5, 14506-0, 17400-3, 21440-3, 30167-1, 38372-9, 44550-2, 49896-4, 59420-0, 6514-4, 69002-4, 73959-9, 59263-4, 71431-1, 75694-0, 77379-6, 77399-4, 77400-0, 82354-2, 82456-5
	ICD-10	Z12.4, Z11.51
	ICD-9	V76.2, V73.81
	CPT	88142, 88164, 88165, 88166, 88167, 88174, 88175, 88141, 88143, 88147, 88148, 88150, 88152, 88153, 88154, 88155, 88156
		87621, 87624, 87625
	HCPCS (G-Codes)	G0123, G0143, G0144, G0145, G0147, G0148, P3000, G0101, G0124, G0141, P3001, Q0091, G0476
Colorectal Cancer Screening	ICD-10	Z12.11, Z12.12

© 2026 Gurayah AA et al. *JAMA Network Open.*

	ICD-9	V76.51
	CPT	45355, 45378, 45380, 45381, 45382, 45384, 45385, 45388, 45389, 45390, 45391, 45392, 45393, 45331, 45339, 45335, 45337, 45340, 45341, 45342, 45346, 45349, 74263, 81528
	HCPCS (G-Codes)	G0328, G0105, G0121, G0104
	ICD-9 Procedure Codes	45.23, 45.43, 45.42, 45.25, 45.22, 44397, 45.24
	LOINC (FIT/FOBT)	77353-1, 77354-9, 82270, 82274, 29771-3, 12503-9, 14563-1, 14564-9, 2335-8, 27396-1, 27401-9, 27925-7, 56490-6, 56491-4, 57905-2, 58453-2, 80372-6
Prostate Cancer Screening	LOINC (PSA)	2857-1, 35741-8, 83112-3, 19195-7
	HCPCS	G0103
Lung Cancer Screening	CPT	71271
	HCPCS	G0297
	SNOMED	724167008
**CANCER DIAGNOSIS**		
Site	Code Type	Codes
Breast Cancer	ICD-9	174.6, 174.9, 174.1, 174.3, 174.5, 174.0, 174.8, V10.3
	ICD-10	D05, D05.12, D05.11, D05.10, D05.02, D05.01, D05.00, D05.82, D05.81, D05.80, D05.92, D05.91, D05.90, C50, C50.612, C50.611, C50.619, C50.112, C50.111, C50.119, C50.312, C50.311, C50.319, C50.512, C50.511, C50.519, C50.012, C50.011, C50.019, C50.812, C50.811, C50.819, C50.912, C50.911, C50.919, C50.212, C50.211,

© 2026 Gurayah AA et al. *JAMA Network Open.*

		C50.219, C50.412, C50.411, C50.419, Z85.3
Cervical Cancer	ICD-9	180, 180.9, 180.0, 180.1, 180.8
	ICD-10	C53, C53.9, C53.0, C53.1, C53.8
Colorectal Cancer	ICD-9	154.2, 154.3, 153.5, 153.6, 153.4, 153, 153.9, 153.2, 153.0, 154.0, 154.1, 154, 154.8, 153.3, 153.7, 153.1
	ICD-10	C21.1, C21, C21.0, C21.2, C21.8, C18.1, C18.2, C18.0, C18, C18.9, C18.8, C18.6, C18.3, C19, C20, C18.7, C18.5, C18.4, Z85.038, Z85.048
Lung	ICD-10	C34
Prostate	ICD-9	790.93, 185, V10.46
	ICD-10	R97.2, R97.20, C61, Z85.46
	SNOMED	399490008, 254900004, 399068003, 314994000, 459381000124106, 436401000124103, 436431000124106, 444808002, 92308005, 92691004, 712849003, 722081007, 722103009, 461511000124101, 1098981000119101, 93974005, 427492003
**SITE SPECIFIC RESECTIONS**		
Site	Code Type	Codes
Breast	ICD-9	85.44, 85.42, 85.47, 85.43, 85.45, 85.41
	ICD-10	19307, 19240, 19306, 19305, 19200, 19303, 19180, 19304, 19182, 0HTV0ZZ, 0HTU0ZZ
Cervical	ICD-9	58953, 58954, 58956, 57540, 57550, 57555, 68.71, 68.41, 68.51, 58575, 58548, 58570, 58571, 58572, 58573, 58550, 58552, 58553, 58554, 56308, 68.9, 68.79, 68.39, 68.49, 68.59, 68.8, 58240, 51597, 58210, 58951, 58152, 58200, 58260, 58267, 58262, 58263, 58270,

© 2026 Gurayah AA et al. *JAMA Network Open.*

		58290, 58293, 58291, 58292, 58294, 58275
	ICD-10	0UTC0ZZ, 0UTC4ZZ, 0UTC7ZZ, 0UTC8ZZ, 0UT40ZZ, 0UT90ZZ, 0UT94ZZ, 0UT97ZZ, 0UT98ZZ, 0UT9FZZ, V88.01, Z90.710, V88.03, Z90.712, V88.02, Z90.711
Colorectal	ICD-9	44156, 44158, 44157, 44155, 44150, 45.81, 45.82, 45.83
	ICD-10	44211, 44212, 44210, 0DTE0ZZ, 0DTE4ZZ
Prostate	ICD-9	60.6, 60.69, 60.29, 60.62, 55831, 55821, 55810, 55815, 55812, 55801, 55840, 55845, 55842, 60.5, 60.4, 60.3, 60.21, 60.2
	ICD-10	0VT00ZZ, 0VT04ZZ, 0VT07ZZ, 0VT08ZZ
	SNOMED/Other	427985002, 55866, 176258007, 4163F, 41371003, 90470006, 26294005, 67598001, 708919000, 36253005, 176288003, 90199006

© 2026 Gurayah AA et al. *JAMA Network Open.*

Supplemental Table 2. Participant characteristics for the breast cancer screening cohort stratified by healthcare access barrier burden

Characteristic	Overall<br>N = 42,908	0<br>N = 26,914	1<br>N = 7,889	2<br>N = 3,905	3+<br>N = 4,200
**Age at Last Follow Up (Years)**					
Median (Q1, Q3)	60 (52, 67)	62 (54, 69)	59 (51, 66)	56 (50, 63)	55 (48, 62)
**Race and Ethnicity**					
Hispanic	4,442 (10%)	2,560 (9.5%)	865 (11%)	443 (11%)	574 (14%)
Non-Hispanic Black	4,899 (11%)	2,884 (11%)	960 (12%)	483 (12%)	572 (14%)
Non-Hispanic White	30,800 (72%)	19,883 (74%)	5,510 (70%)	2,695 (69%)	2,712 (65%)
Other race or ethnicity[a]	1,708 (4.0%)	990 (3.7%)	344 (4.4%)	186 (4.8%)	188 (4.5%)
Unknown	1,059 (2.5%)	597 (2.2%)	210 (2.7%)	98 (2.5%)	154 (3.7%)
**Marital Status**					
Divorced/Separated/Widowed	11,301 (26%)	6,555 (24%)	2,151 (27%)	1,219 (31%)	1,376 (33%)
Married/Living with Partner	25,665 (60%)	17,002 (63%)	4,494 (57%)	2,067 (53%)	2,102 (50%)
Never Married	5,443 (13%)	3,076 (11%)	1,153 (15%)	567 (15%)	647 (15%)
Unknown	499 (1.2%)	281 (1.0%)	91 (1.2%)	52 (1.3%)	75 (1.8%)
**Annual Income**					
$0-$24,999	6,576 (15%)	3,311 (12%)	1,448 (18%)	858 (22%)	959 (23%)
$25,000-$49,999	6,216 (14%)	3,416 (13%)	1,173 (15%)	638 (16%)	989 (24%)
$50,000-$74,999	5,833 (14%)	3,610 (13%)	1,015 (13%)	545 (14%)	663 (16%)
$75,000-$99,999	5,103 (12%)	3,345 (12%)	937 (12%)	420 (11%)	401 (9.5%)
$100,000 or greater	13,917 (32%)	9,825 (37%)	2,439 (31%)	997 (26%)	656 (16%)
Unknown	5,263 (12%)	3,407 (13%)	877 (11%)	447 (11%)	532 (13%)
**Educational Attainment**					
High School or Less	6,438 (15%)	3,813 (14%)	1,223 (16%)	635 (16%)	767 (18%)
Some College	11,752 (27%)	6,649 (25%)	2,246 (28%)	1,282 (33%)	1,575 (38%)
College	11,949 (28%)	7,750 (29%)	2,183 (28%)	1,017 (26%)	999 (24%)
Advanced Degree	12,256 (29%)	8,392 (31%)	2,135 (27%)	929 (24%)	800 (19%)
Unknown	513 (1.2%)	310 (1.2%)	102 (1.3%)	42 (1.1%)	59 (1.4%)
**Home Ownership**					
Own	29,733 (69%)	20,091 (75%)	5,102 (65%)	2,344 (60%)	2,196 (52%)
Rent	10,354 (24%)	5,413 (20%)	2,206 (28%)	1,215 (31%)	1,520 (36%)
Other/Unknown	2,821 (6.6%)	1,410 (5.2%)	581 (7.4%)	346 (8.9%)	484 (12%)
**Health Insurance**					
Self/Employer Purchased	22,635 (53%)	14,130 (53%)	4,169 (53%)	2,129 (55%)	2,207 (53%)
Medicare	11,728 (27%)	8,325 (31%)	1,875 (24%)	729 (19%)	799 (19%)
Medicaid	5,310 (12%)	2,681 (10.0%)	1,219 (15%)	712 (18%)	698 (17%)
Military/VA	898 (2.1%)	566 (2.1%)	192 (2.4%)	85 (2.2%)	55 (1.3%)
Other	972 (2.3%)	550 (2.0%)	175 (2.2%)	102 (2.6%)	145 (3.5%)
None	1,015 (2.4%)	456 (1.7%)	190 (2.4%)	111 (2.8%)	258 (6.1%)
Unknown	350 (0.8%)	206 (0.8%)	69 (0.9%)	37 (0.9%)	38 (0.9%)
**Employment Status**					
Employed	22,809 (53%)	13,797 (51%)	4,340 (55%)	2,255 (58%)	2,417 (58%)
Retired	10,523 (25%)	8,144 (30%)	1,490 (19%)	503 (13%)	386 (9.2%)
Unable/Out of Work	7,001 (16%)	3,409 (13%)	1,571 (20%)	902 (23%)	1,119 (27%)
Other	2,575 (6.0%)	1,564 (5.8%)	488 (6.2%)	245 (6.3%)	278 (6.6%)
**Census Region of Residence**					
Northeast	13,619 (32%)	8,704 (32%)	2,568 (33%)	1,184 (30%)	1,163 (28%)
Midwest	16,212 (38%)	10,502 (39%)	2,859 (36%)	1,451 (37%)	1,400 (33%)
South	5,318 (12%)	3,212 (12%)	946 (12%)	500 (13%)	660 (16%)
West	<7,750 (18%)	<4,490	<1,514	<770	<980
Other	<20 (<1%)	<20	<20	<20	<20

© 2026 Gurayah AA et al. *JAMA Network Open.*

[a]Other race and ethnicity includes participants who self-identified as Non-Hispanic Asian or Pacific Islander, Non-Hispanic American Indian or Alaska Native, Non-Hispanic Middle Eastern or North African, or as belonging to two or more racial groups.

Per the data and statistics dissemination policy of the All of Us Research Program, all participant counts less than 20 individuals have been suppressed.

© 2026 Gurayah AA et al. *JAMA Network Open.*

Supplemental Table 3. Participant characteristics for the cervical cancer screening cohort stratified by healthcare access barrier burden

Characteristic	Overall<br>N = 45,791	0<br>N = 23,004	1<br>N = 9,845	2<br>N = 5,820	3+<br>N = 7,122
**Age at Last Follow Up (Years)**					
Median (Q1, Q3)	46 (35, 56)	49 (38, 58)	44 (34, 55)	43 (33, 54)	42 (32, 52)
**Race and Ethnicity**					
Hispanic	29,716 (65%)	15,250 (66%)	6,411 (65%)	3,732 (64%)	4,323 (61%)
Non-Hispanic Black	7,324 (16%)	3,526 (15%)	1,613 (16%)	916 (16%)	1,269 (18%)
Non-Hispanic White	4,863 (11%)	2,407 (10%)	984 (10.0%)	622 (11%)	850 (12%)
Other race or ethnicity[a]	2,927 (6.4%)	1,401 (6.1%)	641 (6.5%)	419 (7.2%)	466 (6.5%)
Unknown	961 (2.1%)	420 (1.8%)	196 (2.0%)	131 (2.3%)	214 (3.0%)
**Marital Status**					
Divorced/Separated/Widowed	7,445 (16%)	3,578 (16%)	1,528 (16%)	1,000 (17%)	1,339 (19%)
Married/Living with Partner	26,290 (57%)	14,212 (62%)	5,469 (56%)	3,035 (52%)	3,574 (50%)
Never Married	11,426 (25%)	4,906 (21%)	2,732 (28%)	1,706 (29%)	2,082 (29%)
Unknown	630 (1.4%)	308 (1.3%)	116 (1.2%)	79 (1.4%)	127 (1.8%)
**Annual Income**					
$0-$24,999	7,986 (17%)	3,185 (14%)	1,809 (18%)	1,256 (22%)	1,736 (24%)
$25,000-$49,999	7,332 (16%)	2,867 (12%)	1,609 (16%)	1,062 (18%)	1,794 (25%)
$50,000-$74,999	5,807 (13%)	2,666 (12%)	1,267 (13%)	788 (14%)	1,086 (15%)
$75,000-$99,999	4,901 (11%)	2,508 (11%)	1,078 (11%)	657 (11%)	658 (9.2%)
$100,000 or greater	14,252 (31%)	8,890 (39%)	3,003 (31%)	1,391 (24%)	968 (14%)
Unknown	5,513 (12%)	2,888 (13%)	1,079 (11%)	666 (11%)	880 (12%)
**Educational Attainment**					
High School or Less	7,679 (17%)	3,656 (16%)	1,583 (16%)	997 (17%)	1,443 (20%)
Some College	12,209 (27%)	5,300 (23%)	2,643 (27%)	1,742 (30%)	2,524 (35%)
College	13,677 (30%)	7,044 (31%)	2,989 (30%)	1,753 (30%)	1,891 (27%)
Advanced Degree	11,665 (25%)	6,718 (29%)	2,512 (26%)	1,264 (22%)	1,171 (16%)
Unknown	561 (1.2%)	286 (1.2%)	118 (1.2%)	64 (1.1%)	93 (1.3%)
**Home Ownership**					
Own	24,073 (53%)	13,843 (60%)	4,882 (50%)	2,641 (45%)	2,707 (38%)
Rent	16,826 (37%)	7,182 (31%)	3,860 (39%)	2,445 (42%)	3,339 (47%)
Other/Unknown	4,892 (11%)	1,979 (8.6%)	1,103 (11%)	734 (13%)	1,076 (15%)
**Health Insurance**					
Self/Employer Purchased	29,997 (66%)	16,126 (70%)	6,298 (64%)	3,527 (61%)	4,046 (57%)
Medicare	2,779 (6.1%)	1,255 (5.5%)	600 (6.1%)	394 (6.8%)	530 (7.4%)
Medicaid	8,622 (19%)	3,651 (16%)	2,029 (21%)	1,347 (23%)	1,595 (22%)
Military/VA	983 (2.1%)	540 (2.3%)	255 (2.6%)	104 (1.8%)	84 (1.2%)
Other	1,220 (2.7%)	527 (2.3%)	251 (2.5%)	179 (3.1%)	263 (3.7%)
None	1,747 (3.8%)	701 (3.0%)	325 (3.3%)	209 (3.6%)	512 (7.2%)
Unknown	443 (1.0%)	204 (0.9%)	87 (0.9%)	60 (1.0%)	92 (1.3%)
**Employment Status**					
Employed	32,043 (70%)	16,527 (72%)	6,830 (69%)	3,941 (68%)	4,745 (67%)
Retired	1,519 (3.3%)	1,034 (4.5%)	275 (2.8%)	112 (1.9%)	98 (1.4%)
Unable/Out of Work	7,661 (17%)	3,205 (14%)	1,711 (17%)	1,177 (20%)	1,568 (22%)
Other	4,568 (10.0%)	2,238 (9.7%)	1,029 (10%)	590 (10%)	711 (10.0%)
**Census Region of Residence**					
Northeast	14,100 (31%)	7,157 (31%)	3,211 (33%)	1,781 (31%)	1,951 (27%)
Midwest	15,367 (34%)	8,130 (35%)	3,186 (32%)	1,968 (34%)	2,083 (29%)
South	6,226 (14%)	2,919 (13%)	1,244 (13%)	782 (13%)	1,281 (18%)
West	<10,090 (22%)	<4,800	<2,210	<1,290	<1,810
Other	<20 (<1%)	<20	<20	<20	<20

© 2026 Gurayah AA et al. *JAMA Network Open.*

[a]Other race and ethnicity includes participants who self-identified as Non-Hispanic Asian or Pacific Islander, Non-Hispanic American Indian or Alaska Native, Non-Hispanic Middle Eastern or North African, or as belonging to two or more racial groups.

Per the data and statistics dissemination policy of the All of Us Research Program, all participant counts less than 20 individuals have been suppressed.

© 2026 Gurayah AA et al. *JAMA Network Open*.

Supplemental Table 4. Participant characteristics for the colorectal cancer screening cohort stratified by healthcare access barrier burden

Characteristic	Overall<br>N = 55,986	0<br>N = 40,373	1<br>N = 8,590	2<br>N = 3,608	3+<br>N = 3,415
**Age at Last Follow Up (Years)**					
Median (Q1, Q3)	66 (57, 73)	67 (59, 74)	63 (55, 70)	59 (52, 66)	58 (51, 64)
**Sex at Birth**					
Female	35,208 (63%)	24,104 (60%)	5,877 (68%)	2,649 (73%)	2,578 (75%)
Male	20,356 (36%)	15,988 (40%)	2,645 (31%)	922 (26%)	801 (23%)
Other or Unknown	422 (0.7%)	281 (0.7%)	68 (0.7%)	37 (1.0%)	36 (1.0%)
**Race and Ethnicity**					
Hispanic	3,932 (7.0%)	2,518 (6.2%)	713 (8.3%)	324 (9.0%)	377 (11%)
Non-Hispanic Black	5,333 (9.5%)	3,313 (8.2%)	965 (11%)	499 (14%)	556 (16%)
Non-Hispanic White	43,166 (77%)	32,096 (79%)	6,326 (74%)	2,528 (70%)	2,216 (65%)
Other race or ethnicity[a]	2,078 (3.7%)	1,398 (3.5%)	373 (4.3%)	158 (4.4%)	149 (4.4%)
Unknown	1,477 (2.6%)	1,048 (2.6%)	213 (2.5%)	99 (2.7%)	117 (3.4%)
**Marital Status**					
Divorced/Separated/Widowed	13,055 (23%)	8,705 (22%)	2,215 (26%)	1,054 (29%)	1,081 (32%)
Married/Living with Partner	36,021 (64%)	27,310 (68%)	5,061 (59%)	1,940 (54%)	1,710 (50%)
Never Married	6,302 (11%)	3,974 (9.8%)	1,210 (14%)	559 (15%)	559 (16%)
Unknown	608 (1.1%)	384 (1.0%)	104 (1.2%)	55 (1.5%)	65 (1.9%)
**Annual Income**					
$0-$24,999	6,654 (12%)	3,726 (9.2%)	1,383 (16%)	740 (21%)	805 (24%)
$25,000-$49,999	7,602 (14%)	4,955 (12%)	1,289 (15%)	590 (16%)	768 (22%)
$50,000-$74,999	7,706 (14%)	5,551 (14%)	1,153 (13%)	502 (14%)	500 (15%)
$75,000-$99,999	6,955 (12%)	5,211 (13%)	990 (12%)	408 (11%)	346 (10%)
$100,000 or greater	20,578 (37%)	16,271 (40%)	2,818 (33%)	946 (26%)	543 (16%)
Unknown	6,491 (12%)	4,659 (12%)	957 (11%)	422 (12%)	453 (13%)
**Educational Attainment**					
High School or Less	7,170 (13%)	4,756 (12%)	1,181 (14%)	595 (16%)	638 (19%)
Some College	13,578 (24%)	8,934 (22%)	2,314 (27%)	1,100 (30%)	1,230 (36%)
College	15,748 (28%)	11,512 (29%)	2,420 (28%)	968 (27%)	848 (25%)
Advanced Degree	18,879 (34%)	14,753 (37%)	2,582 (30%)	898 (25%)	646 (19%)
Unknown	611 (1.1%)	418 (1.0%)	93 (1.1%)	47 (1.3%)	53 (1.6%)
**Home Ownership**					
Own	42,043 (75%)	31,983 (79%)	5,936 (69%)	2,256 (63%)	1,868 (55%)
Rent	11,099 (20%)	6,680 (17%)	2,152 (25%)	1,063 (29%)	1,204 (35%)
Other/Unknown	2,844 (5.1%)	1,710 (4.2%)	502 (5.8%)	289 (8.0%)	343 (10%)
**Health Insurance**					
Self/Employer Purchased	23,681 (42%)	16,286 (40%)	3,910 (46%)	1,827 (51%)	1,658 (49%)
Medicare	23,485 (42%)	18,813 (47%)	2,970 (35%)	897 (25%)	805 (24%)
Medicaid	4,316 (7.7%)	2,366 (5.9%)	932 (11%)	525 (15%)	493 (14%)
Military/VA	1,894 (3.4%)	1,372 (3.4%)	312 (3.6%)	130 (3.6%)	80 (2.3%)
Other	1,006 (1.8%)	629 (1.6%)	169 (2.0%)	87 (2.4%)	121 (3.5%)
None	1,096 (2.0%)	566 (1.4%)	206 (2.4%)	103 (2.9%)	221 (6.5%)
Unknown	508 (0.9%)	341 (0.8%)	91 (1.1%)	39 (1.1%)	37 (1.1%)
**Employment Status**					
Employed	24,869 (44%)	16,894 (42%)	4,219 (49%)	1,940 (54%)	1,816 (53%)
Retired	22,302 (40%)	18,470 (46%)	2,626 (31%)	725 (20%)	481 (14%)
Unable/Out of Work	6,737 (12%)	3,666 (9.1%)	1,376 (16%)	769 (21%)	926 (27%)
Other	2,078 (3.7%)	1,343 (3.3%)	369 (4.3%)	174 (4.8%)	192 (5.6%)
**Census Region of Residence**					
Northeast	20,602 (37%)	14,774 (37%)	3,239 (38%)	1,333 (37%)	1,256 (37%)
Midwest	22,333 (40%)	16,291 (40%)	3,324 (39%)	1,459 (40%)	1,259 (37%)
South	6,015 (11%)	4,102 (10%)	929 (11%)	432 (12%)	552 (16%)
West	7,004 (13%)	<5,190	<1,100	<390	<350

© 2026 Gurayah AA et al. *JAMA Network Open.*

Other	32 (<0.1%)	<30	<20	<20	<20

[a]Other race and ethnicity includes participants who self-identified as Non-Hispanic Asian or Pacific Islander, Non-Hispanic American Indian or Alaska Native, Non-Hispanic Middle Eastern or North African, or as belonging to two or more racial groups.

Per the data and statistics dissemination policy of the All of Us Research Program, all participant counts less than 20 individuals have been suppressed.

© 2026 Gurayah AA et al. *JAMA Network Open.*

Supplemental Table 5. Participant characteristics for the lung cancer screening cohort stratified by healthcare access barrier burden

Characteristic	Overall<br>N = 3,358	0<br>N = 2,149	1<br>N = 612	2<br>N = 275	3+<br>N = 322
**Age at Last Follow Up (Years)**					
Median (Q1, Q3)	66 (59, 72)	68 (61, 73)	65 (58, 70)	62 (57, 68)	60 (55, 65)
**Sex at Birth**					
Female	1,796 (53%)	1,048 (49%)	379 (62%)	155 (56%)	214 (66%)
Male	1,532 (46%)	<1090	<230	<120	<110
Other or Unknown	30 (0.8%)	<20	<20	<20	<20
**Race and Ethnicity**					
Hispanic	143 (4.3%)	<100	<20	<20	<20
Non-Hispanic Black	275 (8.2%)	<180	<60	<30	<20
Non-Hispanic White	2,777 (83%)	1,765 (82%)	525 (86%)	216 (79%)	271 (84%)
Other race or ethnicity[a]	72 (2.1%)	<50	<20	<20	<20
Unknown	91 (2.7%)	<70	<20	<20	<20
**Marital Status**					
Divorced/Separated/Widowed	1,207 (36%)	713 (33%)	240 (39%)	115 (42%)	139 (43%)
Married/Living with Partner	1,683 (50%)	1,165 (54%)	265 (43%)	111 (40%)	142 (44%)
Never Married	435 (13%)	<260	<110	<50	<40
Unknown	33 (1.0%)	<20	<20	<20	<20
**Annual Income**					
$0-$24,999	848 (25%)	452 (21%)	187 (31%)	112 (41%)	97 (30%)
$25,000-$49,999	749 (22%)	443 (21%)	152 (25%)	53 (19%)	101 (31%)
$50,000-$74,999	518 (15%)	359 (17%)	83 (14%)	32 (12%)	44 (14%)
$75,000-$99,999	383 (11%)	272 (13%)	61 (10.0%)	23 (8.4%)	27 (8.4%)
$100,000 or greater	546 (16%)	412 (19%)	80 (13%)	26 (9.5%)	28 (8.7%)
Unknown	314 (9.4%)	211 (9.8%)	49 (8.0%)	29 (11%)	25 (7.8%)
**Educational Attainment**					
High School or Less	741 (22%)	466 (22%)	127 (21%)	78 (28%)	70 (22%)
Some College	1,415 (42%)	860 (40%)	268 (44%)	120 (44%)	167 (52%)
College	683 (20%)	456 (21%)	122 (20%)	49 (18%)	56 (17%)
Advanced Degree	477 (14%)	337 (16%)	90 (15%)	23 (8.4%)	27 (8.4%)
Unknown	42 (1.3%)	<40	<20	<20	<20
**Home Ownership**					
Own	2,058 (61%)	1,431 (67%)	345 (56%)	127 (46%)	155 (48%)
Rent	1,011 (30%)	562 (26%)	212 (35%)	109 (40%)	128 (40%)
Other/Unknown	289 (8.6%)	156 (7.3%)	55 (9.0%)	39 (14%)	39 (12%)
**Health Insurance**					
Self/Employer Purchased	938 (28%)	557 (26%)	185 (30%)	77 (28%)	119 (37%)
Medicare	1,534 (46%)	1,079 (50%)	248 (41%)	106 (39%)	101 (31%)
Medicaid	515 (15%)	288 (13%)	101 (17%)	61 (22%)	65 (20%)
Military/VA	171 (5.1%)	<130	<30	<20	<20
Other	76 (2.3%)	<50	<20	<20	<20
None	94 (2.8%)	<50	<30	<20	<20
Unknown	30 (0.9%)	<20	<20	<20	<20
**Employment Status**					
Employed	1,144 (34%)	678 (32%)	224 (37%)	91 (33%)	151 (47%)
Retired	1,351 (40%)	1,027 (48%)	201 (33%)	74 (27%)	49 (15%)
Unable/Out of Work	786 (23%)	<410	<170	<110	<120
Other	77 (2.3%)	<50	<30	<20	<20
**Census Region of Residence**					
Northeast	1,137 (34%)	712 (33%)	219 (36%)	90 (33%)	116 (36%)
Midwest	1,209 (36%)	803 (37%)	215 (35%)	86 (31%)	105 (33%)
South	354 (11%)	233 (11%)	58 (9.5%)	25 (9.1%)	38 (12%)
West	<660 (20%)	<410	<130	<80	<70

© 2026 Gurayah AA et al. *JAMA Network Open.*

Characteristic	Overall<br>N = 3,358	0<br>N = 2,149	1<br>N = 612	2<br>N = 275	3+<br>N = 322
Other	<20 (<1%)	<20	<20	<20	<20
**Smoking Status**					
Current	968 (29%)	545 (25%)	205 (33%)	124 (37%)	136 (36%)
Former	2,390 (71%)	1,604 (75%)	407 (67%)	174 (63%)	205 (64%)
**Smoking Pack-Year History**					
Median (Q1, Q3)	37 (26, 50)	38 (27, 50)	35 (25, 45)	37 (27, 50)	35 (26, 46)

[a]Other race and ethnicity includes participants who self-identified as Non-Hispanic Asian or Pacific Islander, Non-Hispanic American Indian or Alaska Native, Non-Hispanic Middle Eastern or North African, or as belonging to two or more racial groups.

Per the data and statistics dissemination policy of the All of Us Research Program, all participant counts less than 20 individuals have been suppressed.

© 2026 Gurayah AA et al. *JAMA Network Open.*

Supplemental Table 6. Participant characteristics for the prostate cancer screening cohort stratified by healthcare access barrier burden

Characteristic	Overall<br>N = 12,648	0<br>N = 9,152	1<br>N = 1,916	2<br>N = 793	3+<br>N = 787
**Age at Last Follow Up (Years)**					
Median (Q1, Q3)	63 (59, 66)	64 (60, 67)	63 (59, 66)	62 (58, 65)	61 (58, 65)
**Race and Ethnicity**					
Hispanic	1,267 (10%)	848 (9.3%)	209 (11%)	95 (12%)	115 (15%)
Non-Hispanic Black	1,170 (9.3%)	744 (8.1%)	227 (12%)	101 (13%)	98 (12%)
Non-Hispanic White	9,351 (74%)	6,955 (76%)	1,358 (71%)	536 (68%)	502 (64%)
Other race or ethnicity[a]	527 (4.2%)	377 (4.1%)	76 (4.0%)	36 (4.5%)	38 (4.8%)
Unknown	333 (2.6%)	228 (2.5%)	46 (2.4%)	25 (3.2%)	34 (4.3%)
**Marital Status**					
Divorced/Separated/Widowed	2,330 (18%)	1,451 (16%)	436 (23%)	211 (27%)	232 (29%)
Married/Living with Partner	8,553 (68%)	6,567 (72%)	1,152 (60%)	446 (56%)	388 (49%)
Never Married	1,609 (13%)	<1,040	<310	<130	<150
Unknown	156 (1.2%)	<110	<30	<20	<30
**Annual Income**					
$0-$24,999	1,968 (16%)	1,107 (12%)	419 (22%)	189 (24%)	253 (32%)
$25,000-$49,999	1,378 (11%)	873 (9.5%)	240 (13%)	125 (16%)	140 (18%)
$50,000-$74,999	1,358 (11%)	964 (11%)	218 (11%)	82 (10%)	94 (12%)
$75,000-$99,999	1,301 (10%)	979 (11%)	169 (8.8%)	88 (11%)	65 (8.3%)
$100,000 or greater	5,133 (41%)	4,177 (46%)	644 (34%)	200 (25%)	112 (14%)
Unknown	1,510 (12%)	1,052 (11%)	226 (12%)	109 (14%)	123 (16%)
**Educational Attainment**					
High School or Less	2,157 (17%)	1,360 (15%)	399 (21%)	183 (23%)	215 (27%)
Some College	3,128 (25%)	2,082 (23%)	534 (28%)	247 (31%)	265 (34%)
College	3,381 (27%)	2,550 (28%)	471 (25%)	182 (23%)	178 (23%)
Advanced Degree	3,840 (30%)	<3,070	<500	<170	<120
Unknown	142 (1.1%)	<100	<20	<20	<20
**Home Ownership**					
Own	8,826 (70%)	6,804 (74%)	1,190 (62%)	459 (58%)	373 (47%)
Rent	2,927 (23%)	1,809 (20%)	557 (29%)	253 (32%)	308 (39%)
Other/Unknown	895 (7.1%)	539 (5.9%)	169 (8.8%)	81 (10%)	106 (13%)
**Health Insurance**					
Self/Employer Purchased	6,448 (51%)	4,937 (54%)	884 (46%)	353 (45%)	274 (35%)
Medicare	3,025 (24%)	2,212 (24%)	451 (24%)	174 (22%)	188 (24%)
Medicaid	1,436 (11%)	870 (9.5%)	296 (15%)	114 (14%)	156 (20%)
Military/VA	867 (6.9%)	638 (7.0%)	128 (6.7%)	63 (7.9%)	38 (4.8%)
Other	273 (2.2%)	161 (1.8%)	43 (2.2%)	31 (3.9%)	38 (4.8%)
None	474 (3.7%)	<260	<100	<50	<90
Unknown	125 (1.0%)	<90	<30	<20	<20
**Employment Status**					
Employed	6,607 (52%)	4,940 (54%)	952 (50%)	392 (49%)	323 (41%)
Retired	3,507 (28%)	2,723 (30%)	480 (25%)	162 (20%)	142 (18%)
Unable/Out of Work	2,336 (18%)	<1,370	<450	<230	<310
Other	198 (1.6%)	<130	<40	<20	<30
**Census Region of Residence**					
Northeast	3,546 (28%)	2,636 (29%)	518 (27%)	193 (24%)	199 (25%)
Midwest	4,173 (33%)	3,132 (34%)	604 (32%)	243 (31%)	194 (25%)
South	1,839 (15%)	1,271 (14%)	271 (14%)	137 (17%)	160 (20%)
West	<3,090 (24%)	<2,110	<530	<230	<240
Other	<20 (<1%)	<20	<20	<20	<20

[a]Other race and ethnicity includes participants who self-identified as Non-Hispanic Asian or Pacific Islander, Non-Hispanic American Indian or Alaska Native, Non-Hispanic Middle Eastern or North African, or as belonging to two or more racial groups.

© 2026 Gurayah AA et al. *JAMA Network Open.*

Per the data and statistics dissemination policy of the All of Us Research Program, all participant counts less than 20 individuals have been suppressed.

© 2026 Gurayah AA et al. *JAMA Network Open.*

Supplemental Table 7. Proportion of *All of Us* participants eligible for cancer screening who responded that this barrier had led them to delay receiving healthcare

Healthcare Access Barrier	Breast	Cervical	Colorectal	Lung	Prostate
Nervous about seeing a healthcare provider	12%	18%	8.4%	11%	7.6%
Could not get childcare	1.9%	6.0%	1.0%	0.7%	0.5%
Live in a rural area and distance is too far	3.6%	4.6%	2.4%	4.5%	3.1%
Provide care to an adult and could not leave him/her	2.8%	2.7%	2.1%	2.5%	1.7%
Unable to get time off of work	8.9%	17%	5.8%	5.9%	4.7%
Did not have transportation	7.7%	10%	5.5%	10%	6.9%
Could not afford co-pay	8.2%	12%	5.4%	8.3%	5.7%
Deductible was too high/could not afford deductible	10%	14%	6.8%	9.2%	7.0%
Had to pay out of pocket for some or all of the procedure	17%	21%	13%	16%	13%

© 2026 Gurayah AA et al. *JAMA Network Open.*

Supplemental Table 8. Multivariable-adjusted odds ratios (OR) and 95% confidence intervals (95% CI) for adherence with USPSTF cancer screening recommendations by self-reported barriers to healthcare and barrier burden using 25% extended time windows for assessment of screening adherence

**Reason for Delaying Care**	**Breast**		**Cervical**		**Colorectal**		**Lung**		**Prostate**	
	Model 1[a]	Model 2[b]	Model 1[a]	Model 2[b]	Model 1[a]	Model 2[b]	Model 1[a]	Model 2[b]	Model 1[a]	Model 2[b]
**Barrier Burden**										
0	Ref.	Ref.	Ref.	Ref.	Ref.	Ref.	Ref.	Ref.	Ref.	Ref.
1	0.81 (0.77-0.85)	0.86 (0.81-0.90)	0.93 (0.88-0.98)	0.97 (0.91-1.02)	0.91 (0.86-0.96)	0.93 (0.88-0.98)	1.01 (0.79-1.28)	0.98 (0.76-1.26)	0.84 (0.76-0.93)	0.92 (0.82-1.02)
2	0.74 (0.70-0.80)	0.80 (0.75-0.86)	0.84 (0.79-0.90)	0.89 (0.83-0.95)	0.85 (0.79-0.92)	0.86 (0.79-0.93)	0.98 (0.69-1.36)	0.96 (0.67-1.36)	0.80 (0.69-0.93)	0.89 (0.76-1.04)
3+	0.61 (0.57-0.65)	0.72 (0.67-0.77)	0.69 (0.65-0.74)	0.78 (0.73-0.83)	0.80 (0.74-0.87)	0.83 (0.77-0.90)	0.75 (0.52-1.05)	0.69 (0.48-0.98)	0.63 (0.54-0.73)	0.75 (0.64-0.88)
**Nervous about seeing healthcare provider**	0.74 (0.70-0.79)	0.79 (0.74-0.84)	0.84 (0.79-0.88)	0.88 (0.84-0.94)	0.93 (0.87-1.00)	0.95 (0.89-1.02)	0.88 (0.64-1.18)	0.86 (0.62-1.17)	0.81 (0.71-0.93)	0.89 (0.78-1.03)
**Couldn't get childcare**	0.96 (0.83-1.10)	1.06 (0.91-1.23)	0.85 (0.78-0.93)	0.98 (0.89-1.07)	0.81 (0.66-1.00)	0.92 (0.74-1.13)	1.36 (0.44-3.45)	1.34 (0.43-3.51)	0.56 (0.31-0.97)	0.73 (0.40-1.27)
**Live in rural area and distance is too far**	0.53 (0.47-0.58)	0.69 (0.61-0.77)	0.62 (0.55-0.69)	0.77 (0.69-0.87)	0.71 (0.63-0.81)	0.77 (0.68-0.87)	0.78 (0.47-1.24)	0.77 (0.45-1.25)	0.75 (0.61-0.92)	0.87 (0.70-1.08)
**Provide care to an adult and could not leave him/her**	0.71 (0.63-0.80)	0.85 (0.75-0.96)	0.66 (0.58-0.76)	0.82 (0.71-0.95)	0.81 (0.71-0.93)	0.86 (0.75-0.99)	0.71 (0.35-1.29)	0.80 (0.39-1.50)	0.72 (0.54-0.95)	0.85 (0.63-1.13)
**Couldn't get time off work**	0.94 (0.88-1.01)	0.88 (0.82-0.95)	0.98 (0.92-1.03)	0.93 (0.88-0.99)	0.95 (0.88-1.04)	0.95 (0.87-1.03)	0.84 (0.54-1.25)	0.78 (0.50-1.20)	0.80 (0.67-0.95)	0.87 (0.72-1.03)
**Didn't have transportation**	0.56 (0.52-0.60)	0.82 (0.75-0.88)	0.65 (0.61-0.70)	0.89 (0.82-0.97)	0.82 (0.76-0.90)	0.91 (0.83-1.00)	0.83 (0.59-1.13)	0.78 (0.54-1.09)	0.60 (0.52-0.70)	0.77 (0.66-0.90)
**Couldn't afford the copay**	0.68 (0.63-0.73)	0.82 (0.76-0.89)	0.76 (0.71-0.81)	0.84 (0.79-0.90)	0.91 (0.84-0.99)	0.96 (0.88-1.04)	0.79 (0.55-1.11)	0.76 (0.52-1.08)	0.72 (0.62-0.85)	0.92 (0.78-1.08)
**Deductible was too high**	0.78 (0.73-0.83)	0.81 (0.76-0.87)	0.85 (0.80-0.91)	0.85 (0.80-0.91)	0.86 (0.80-0.93)	0.84 (0.78-0.91)	0.86 (0.62-1.19)	0.85 (0.60-1.19)	0.76 (0.66-0.87)	0.87 (0.75-1.00)
**Had to pay out of pocket for some/all of procedure**	0.77 (0.73-0.81)	0.79 (0.74-0.83)	0.84 (0.80-0.89)	0.84 (0.80-0.89)	0.87 (0.82-0.92)	0.86 (0.81-0.91)	0.94 (0.73-1.20)	0.93 (0.71-1.20)	0.86 (0.78-0.96)	0.90 (0.81-1.01)

[a]Model 1 includes age at last follow-up, sex at birth (colorectal and lung only), smoking status (current vs former; lung only), pack-year history (lung only).

© 2026 Gurayah AA et al. *JAMA Network Open.*

[b]Model 2 includes age at last follow-up, sex at birth (colorectal and lung only), smoking status (current vs former; lung only), pack-year history (lung only), self-identified race/ethnicity, annual income, educational attainment, employment status, health insurance status and type, marital status, and census region of residence.

© 2026 Gurayah AA et al. *JAMA Network Open.*

Supplemental Table 9. Multivariable-adjusted odds ratios (OR) and 95% confidence intervals (95% CI) for adherence with USPSTF cancer screening recommendations by self-reported barriers to healthcare and barrier burden applying inverse probability of selection weights

Reason for Delaying Care	Breast		Cervical		Colorectal		Lung		Prostate	
	Model 1[a]	Model 2[b]	Model 1[a]	Model 2[b]	Model 1[a]	Model 2[b]	Model 1[a]	Model 2[b]	Model 1[a]	Model 2[b]
**Barrier Burden**										
0	Ref.	Ref.	Ref.	Ref.	Ref.	Ref.	Ref.	Ref.	Ref.	Ref.
1	0.84 (0.79-0.88)	0.87 (0.83-0.92)	0.93 (0.88-0.98)	0.96 (0.90-1.01)	0.93 (0.88-0.98)	0.93 (0.89-0.99)	0.87 (0.65-1.16)	0.85 (0.63-1.15)	0.80 (0.72-0.90)	0.89 (0.79-1.00)
2	0.75 (0.70-0.80)	0.79 (0.74-0.85)	0.83 (0.78-0.89)	0.87 (0.81-0.94)	0.84 (0.78-0.91)	0.85 (0.79-0.92)	0.80 (0.53-1.21)	0.83 (0.53-1.30)	0.84 (0.71-1.00)	0.94 (0.79-1.12)
3+	0.62 (0.58-0.67)	0.72 (0.67-0.78)	0.73 (0.68-0.78)	0.80 (0.74-0.86)	0.76 (0.70-0.82)	0.80 (0.73-0.86)	0.73 (0.49-1.09)	0.71 (0.47-1.06)	0.66 (0.56-0.79)	0.75 (0.63-0.90)
**Nervous about seeing healthcare provider**	0.76 (0.71-0.80)	0.79 (0.74-0.84)	0.83 (0.78-0.88)	0.87 (0.82-0.92)	0.89 (0.83-0.95)	0.90 (0.85-0.97)	0.94 (0.65-1.35)	0.93 (0.63-1.36)	0.84 (0.72-0.97)	0.92 (0.78-1.07)
**Couldn't get childcare**	1.01 (0.87-1.17)	1.10 (0.94-1.30)	0.89 (0.81-0.98)	1.00 (0.91-1.11)	0.96 (0.79-1.17)	1.08 (0.88-1.32)	1.13 (0.32-4.00)	1.33 (0.37-4.84)	0.48 (0.25-0.92)	0.60 (0.30-1.19)
**Live in rural area and distance is too far**	0.57 (0.51-0.63)	0.72 (0.63-0.81)	0.65 (0.58-0.73)	0.80 (0.70-0.90)	0.68 (0.61-0.77)	0.75 (0.67-0.85)	0.81 (0.46-1.42)	0.77 (0.41-1.43)	0.81 (0.64-1.02)	0.98 (0.77-1.25)
**Provide care to an adult and could not leave him/her**	0.72 (0.63-0.81)	0.83 (0.74-0.95)	0.69 (0.60-0.80)	0.82 (0.70-0.95)	0.79 (0.70-0.90)	0.86 (0.75-0.98)	0.49 (0.22-1.09)	0.60 (0.27-1.33)	0.68 (0.50-0.93)	0.79 (0.57-1.10)
**Couldn't get time off work**	1.01 (0.94-1.08)	0.93 (0.86-1.00)	1.00 (0.94-1.06)	0.94 (0.88-1.00)	0.94 (0.87-1.02)	0.91 (0.84-0.99)	0.58 (0.35-0.95)	0.55 (0.33-0.93)	0.88 (0.72-1.06)	0.88 (0.72-1.07)
**Didn't have transportation**	0.60 (0.56-0.65)	0.82 (0.76-0.90)	0.71 (0.66-0.77)	0.91 (0.84-1.00)	0.76 (0.70-0.83)	0.86 (0.79-0.93)	0.70 (0.48-1.02)	0.66 (0.44-1.00)	0.62 (0.53-0.74)	0.81 (0.68-0.96)
**Couldn't afford the copay**	0.68 (0.63-0.73)	0.80 (0.74-0.86)	0.79 (0.74-0.85)	0.84 (0.78-0.91)	0.84 (0.77-0.91)	0.89 (0.82-0.97)	0.92 (0.62-1.36)	0.92 (0.61-1.37)	0.80 (0.68-0.96)	0.89 (0.75-1.07)
**Deductible was too high**	0.79 (0.74-0.84)	0.80 (0.75-0.86)	0.86 (0.81-0.92)	0.85 (0.79-0.91)	0.85 (0.79-0.91)	0.84 (0.78-0.91)	0.95 (0.65-1.39)	0.95 (0.64-1.40)	0.83 (0.71-0.97)	0.87 (0.73-1.02)
**Had to pay out of pocket for some/all of procedure**	0.77 (0.73-0.81)	0.77 (0.73-0.81)	0.87 (0.83-0.92)	0.85 (0.80-0.90)	0.86 (0.81-0.90)	0.85 (0.80-0.89)	0.97 (0.72-1.30)	0.96 (0.71-1.30)	0.92 (0.84-1.04)	0.92 (0.81-1.04)

[a]Model 1 includes age at last follow-up, sex at birth (colorectal and lung only), smoking status (current vs former; lung only), pack-year history (lung only).

[b]Model 2 includes age at last follow-up, sex at birth (colorectal and lung only), smoking status (current vs former; lung only), pack-year history (lung only), self-identified race/ethnicity, annual income, educational attainment, employment status, health insurance status and type, marital status, and census region of residence

© 2026 Gurayah AA et al. *JAMA Network Open.*

© 2026 Gurayah AA et al. *JAMA Network Open.*

Supplemental Figure 1. Flowchart for identification of the breast cancer screening cohort

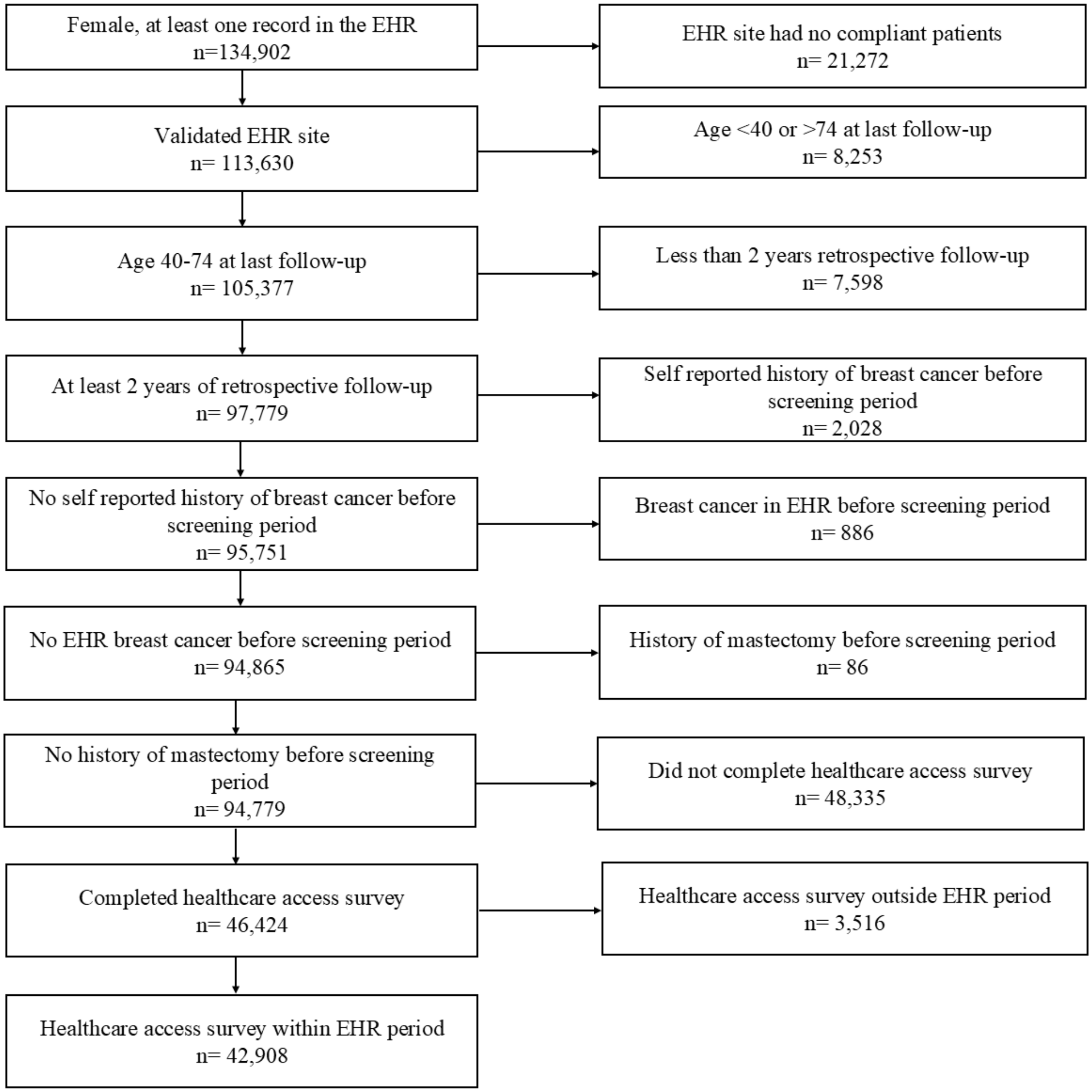

© 2026 Gurayah AA et al. *JAMA Network Open.*

Supplemental Figure 2. Flowchart for identification of the cervical cancer screening cohort

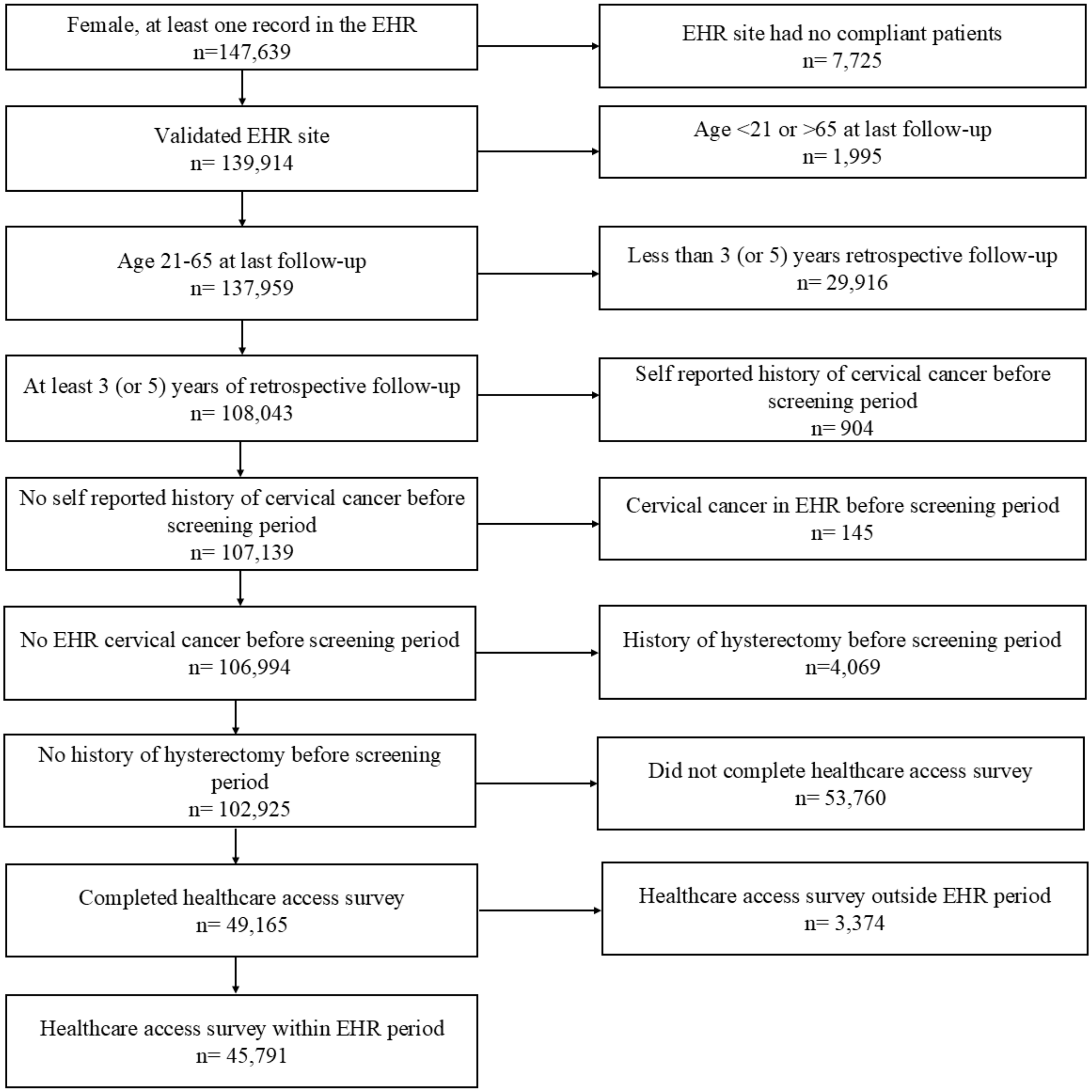

© 2026 Gurayah AA et al. *JAMA Network Open.*

Supplemental Figure 3. Flowchart for identification of the colorectal cancer screening cohort

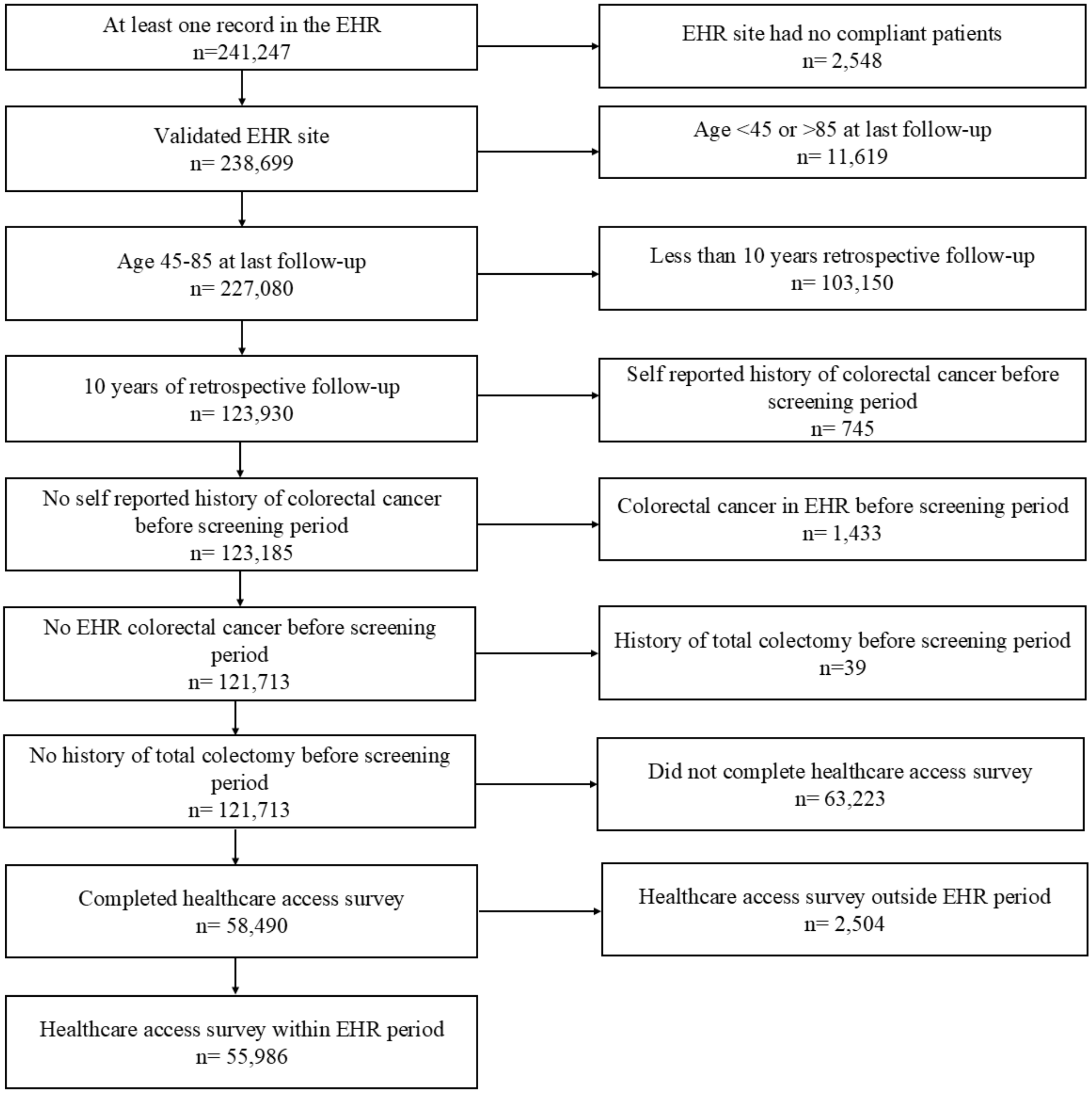

© 2026 Gurayah AA et al. *JAMA Network Open.*

Supplemental Figure 4. Flowchart for identification of the lung cancer screening cohort

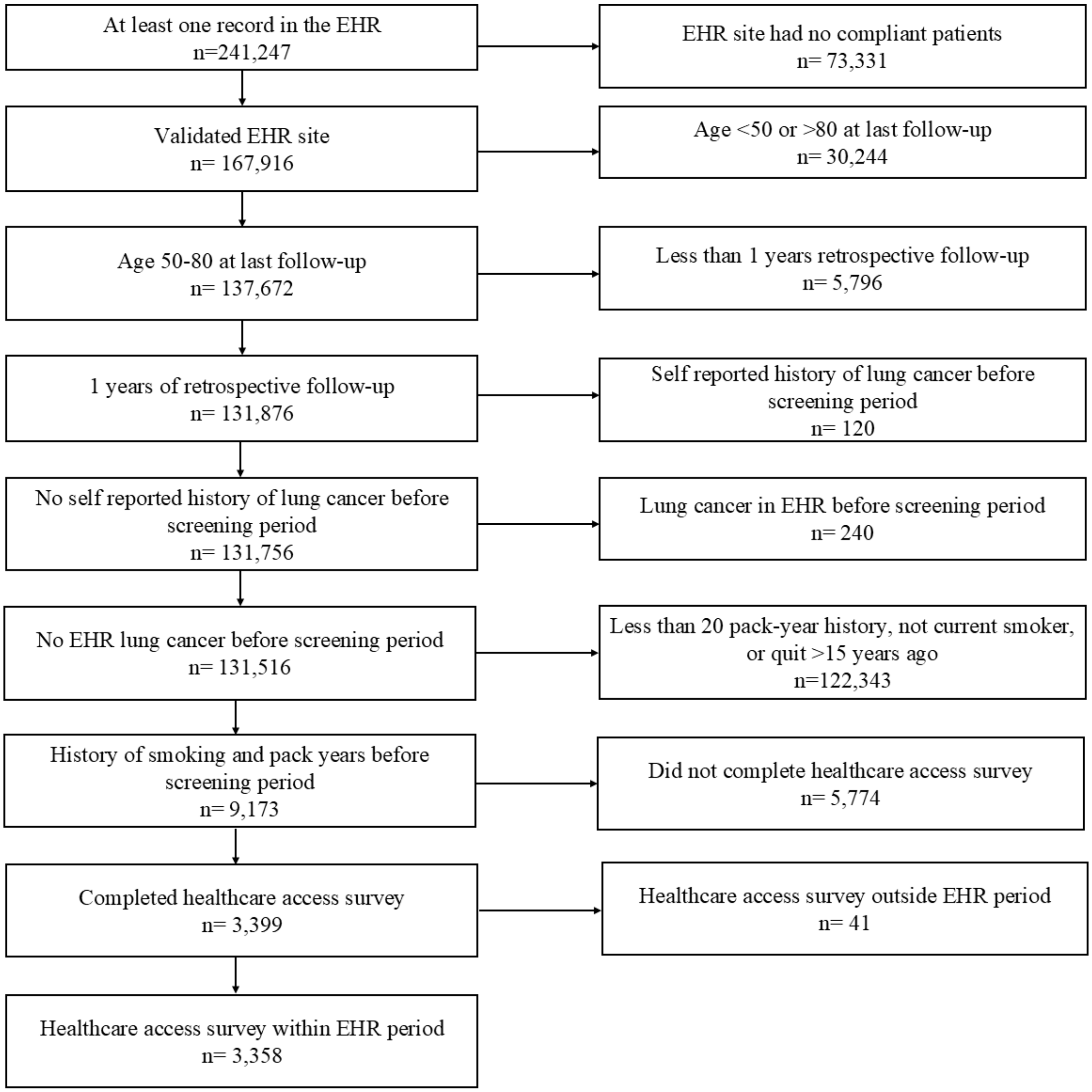

© 2026 Gurayah AA et al. *JAMA Network Open.*

Supplemental Figure 5. Flowchart for identification of the prostate cancer screening cohort

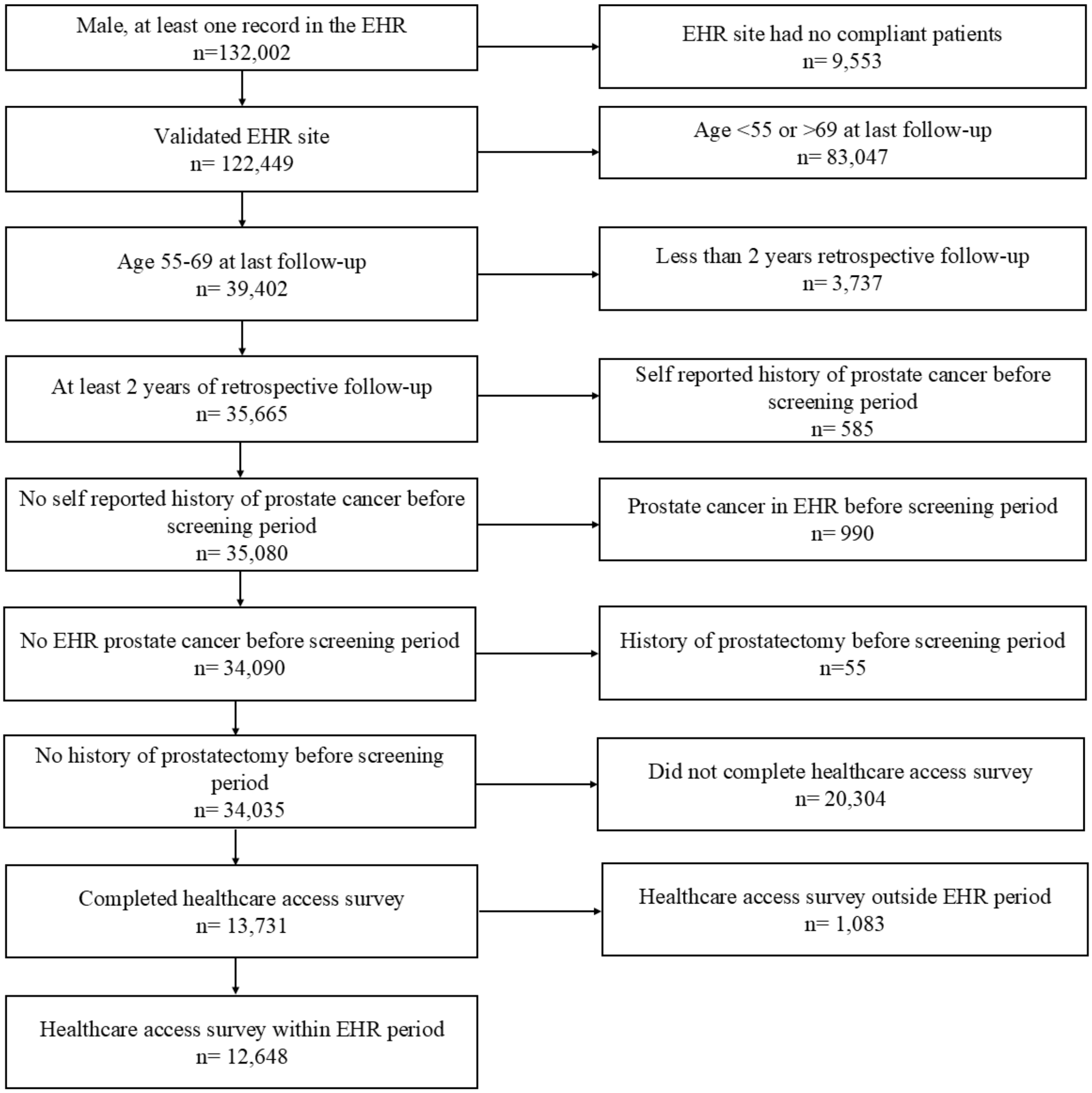

© 2026 Gurayah AA et al. *JAMA Network Open.*

Supplemental Figure 6. Proportion of *All of Us* participants reporting that a barrier has led them to delay receiving care in the past year by cancer screening cohort and adherence with USPSTF guidelines within cohort

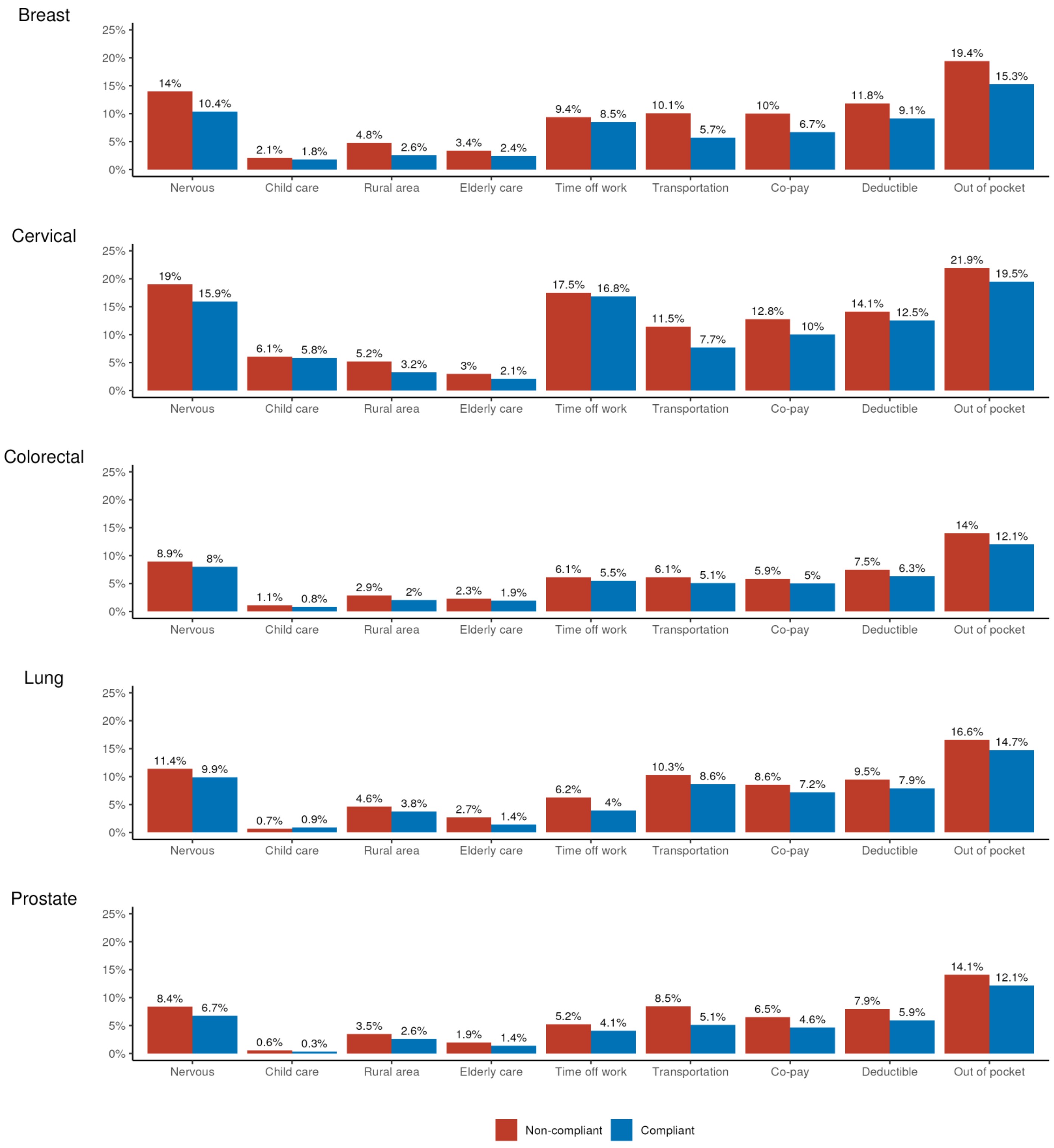

© 2026 Gurayah AA et al. *JAMA Network Open*.
