# Supplementary material for: Barriers to Health Care and Cancer Screening
Source: JAMA Netw Open. 2026 Apr 14;9(4):e267024. doi: 10.1001/jamanetworkopen.2026.7024 (PMC13080545; doi:10.1001/jamanetworkopen.2026.7024)
Supplement: Supplement 2. — Data Sharing Statement [file jamanetwopen-e267024-s002.pdf]

Data Sharing Statement

Gurayah. Barriers to Health Care and Cancer Screening. *JAMA Netw Open*. Published April 14, 2026. doi:10.1001/jamanetworkopen.2026.7024

Data
Data available: No

Additional Information
Explanation for why data not available: The data are made publicly available to registered users through the All of Us Researcher Workbench.
